# COVID-19 – exploring the implications of long-term condition type and extent of multimorbidity on years of life lost: a modelling study

**DOI:** 10.12688/wellcomeopenres.15849.3

**Published:** 2021-03-01

**Authors:** Peter Hanlon, Fergus Chadwick, Anoop Shah, Rachael Wood, Jon Minton, Gerry McCartney, Colin Fischbacher, Frances S. Mair, Dirk Husmeier, Jason Matthiopoulos, David A. McAllister

**Affiliations:** 1 University of Glasgow, Glasgow, UK; 2 University of Edinburgh, Edinburgh, UK; 3 Public Health Scotland, Edinburgh, UK; 4 Scottish Public Health Observatory, NHS Health Scotland, Glasgow, UK

**Keywords:** COVID-19, Coronavirus, Multimorbidity, Epidemiology, noncommunicable diseases

## Abstract

**Background:** COVID-19 is responsible for increasing deaths globally. As most people dying with COVID-19 are older with underlying long-term conditions (LTCs), some speculate that YLL are low. We aim to estimate YLL attributable to COVID-19, before and after adjustment for number/type of LTCs, using the limited data available early in the pandemic.

**Methods:** We first estimated YLL from COVID-19 using WHO life tables, based on published age/sex data from COVID-19 deaths in Italy. We then used aggregate data on number/type of LTCs in a Bayesian model to estimate likely combinations of LTCs among people dying with COVID-19. We used routine UK healthcare data from Scotland and Wales to estimate life expectancy based on age/sex/these combinations of LTCs using Gompertz models from which we then estimate YLL.

**Results:** Using the standard WHO life tables, YLL per COVID-19 death was 14 for men and 12 for women. After adjustment for number and type of LTCs, the mean YLL was slightly lower, but remained high (11.6 and 9.4 years for men and women, respectively). The number and type of LTCs led to wide variability in the estimated YLL at a given age (e.g. at ≥80 years, YLL was >10 years for people with 0 LTCs, and <3 years for people with ≥6).

**Conclusions:** Deaths from COVID-19 represent a substantial burden in terms of per-person YLL, more than a decade, even after adjusting for the typical number and type of LTCs found in people dying of COVID-19. The extent of multimorbidity heavily influences the estimated YLL at a given age. More comprehensive and standardised collection of data (including LTC type, severity, and potential confounders such as socioeconomic-deprivation and care-home status) is needed to optimise YLL estimates for specific populations, and to understand the global burden of COVID-19, and guide policy-making and interventions.

## Introduction

The SARS-CoV-2 pandemic, the virus causing COVID-19, emerged in late 2019 and continues to have substantial impact on populations and healthcare systems throughout the world. This manuscript presents a revised version of an analysis initially conducted in March 2020, at which time Italy, the first European nation to experience a major outbreak of COVID-19, was seeing rapidly escalating numbers of cases and deaths. In the UK, at that time, the initially small number of hospitalisations and deaths were beginning to rise. The analysis sought to estimate the burden of COVID-19 deaths in terms of potential years of life lost (YLL), at a time when individual-level data on COVID-19 deaths was scarce.

When severe, coronavirus disease 2019 (COVID-19) causes acute respiratory failure, often requiring mechanical ventilation ^1^. At the beginning of April 2020, more than 1,200,000 confirmed cases have been reported globally, including 67,000 deaths ^2^. In response to this threat, governments introduced non-pharmaceutical interventions such as physical distancing and the delivery of health services has radically changed, with resources diverted towards the management of COVID-19 and away from their usual activities ^3^. These measures have aimed to limit a surge in cases that risks overwhelming healthcare services ^4^, and have continued and repeated in various forms throughout the world.

Since few health care systems could have responded adequately to the increased need for acute care without these changes, these decisions were in some ways inevitable. However, as societies seek to “return to normal”, decisions about the extent and nature of ongoing measures to limit spread of COVID-19 will be more difficult. These choices will require balancing the likely *direct* effects on mortality from COVID-19 against the likely *indirect* impacts on mortality for other conditions – due, for example, to inadequate access to necessary services for many people with long-term conditions (LTCs), potential reluctance of the public to attend for acute events such as myocardial infarction, or impacts from forced unemployment, loss of income and social isolation. The indirect effects are likely to be complex, most will be downstream, and will require extensive research to be better understood. However, we need to capture the direct effects of COVID-19 as accurately as possible now, via currently available data and methodologies.

In April 2020, most reports of COVID-19 deaths used raw counts ^2^. This may give a distorting picture of the mortality burden, however, as it does not consider how long someone who died from COVID-19 might otherwise have been expected to live. As people dying from COVID-19 are predominantly older and have pre-existing LTCs ^5– 7^, some have speculated that many of these people would have soon died of other causes and that life expectancy may therefore not being greatly impacted ^8, 9^. While multimorbidity, the presence of multiple LTCs, is known to be associated with increased mortality ^10^, people with multimorbidity nonetheless can be expected to live for many years ^11^. Raw counts of deaths may therefore mislead policy-makers and the public, causing them to either over- or under-estimate the total impact of COVID-19 related deaths.

Within epidemiology, there is a standard measure used to account for this difficulty, the years of *potential* life lost (YLL) ^12^. YLL can be expressed per-capita as the average number of years an individual would have been expected to live had they not died of a given cause. The conventional approach to YLL uses data on the age at which deaths occurred combined with typical life expectancy at a given age, to estimate a weighted average of the number of years lost. YLL is used to allow fair comparisons of the health impact of different policies, such as different measures to address the pandemic. However, given the controversial role of multimorbidity in COVID-19 deaths it is also important to calculate YLL additionally considering the effects of the presence of a single LTC or multimorbidity.

Therefore, we propose to quantify the burden of mortality related to COVID-19, both using the conventional age-based YLL measure, and YLL additionally accounting for type and number of underlying LTCs. We draw upon data sources available in April 2020, as this modelling study aimed to estimate the potential YLL at an early stage in the pandemic, when the impact was emerging. It should be noted, however, that events unfolding throughout the pandemic are likely to impact the YLL. Any estimate, particularly in the context of a pandemic, is dependent on what populations are exposed, and to what extent. Updated estimates, taking account of events which transpired in the UK and beyond, are the subject of ongoing collaborative efforts and we have not attempted to model these. Rather, this manuscript provides a detailed and reproducible quantification of YLL using techniques targeting the specific challenges of estimation at the early stages of the pandemic.

## Methods

### WHO standard YLL approach

The standard approach for calculating years of life lost is to apply the distribution of ages among those who died from a specific cause to a standard life-table. For the purposes of international comparison, we opted to use the WHO 2010 Global Burden of Diseases table as the reference ^13^, which presents YLL by age, but not by sex or extent of multimorbidity. This method involves summing the expected years of life remaining from the table according to the number (or for the mean YLL the proportion) of people dying within each age-band. We applied the age distribution of COVID-19 deaths in Italy from published data to estimate the YLL ^14^.

We chose the WHO life tables to allow comparison of the burden of COVID-19 deaths with other conditions in an international context. However these, unlike many national-level life tables, do not stratify by sex. Furthermore our subsequent modelling draws upon data from specific setting based on availability early in the pandemic (namely data on COVID-19 deaths from Italy, and life-expectancy estimates based on data from Wales). Therefore, following comments from academic colleagues via social media, we performed sensitivity analyses using life tables from Italy (2017), United Kingdom (2016–2018) and, for comparison, the United States (2017).

### Overview of modelling to accommodate long-term conditions and multimorbidity

The remainder of the methods describes our approach to estimating YLL accounting for number and type of underlying LTC, along with age and sex. Our modelling comprised three main components: (i) estimating the prevalence of, and correlations between, LTCs among people dying with COVID-19; (ii) modelling UK life expectancy based on age, sex, and each combination of these LTCs separately; and (iii) combining these models to calculate the estimated YLL per death with COVID-19. These are summarised by age-group, sex, and multimorbidity counts (that take into account different combinations of LTCs).

**Figure 1.**
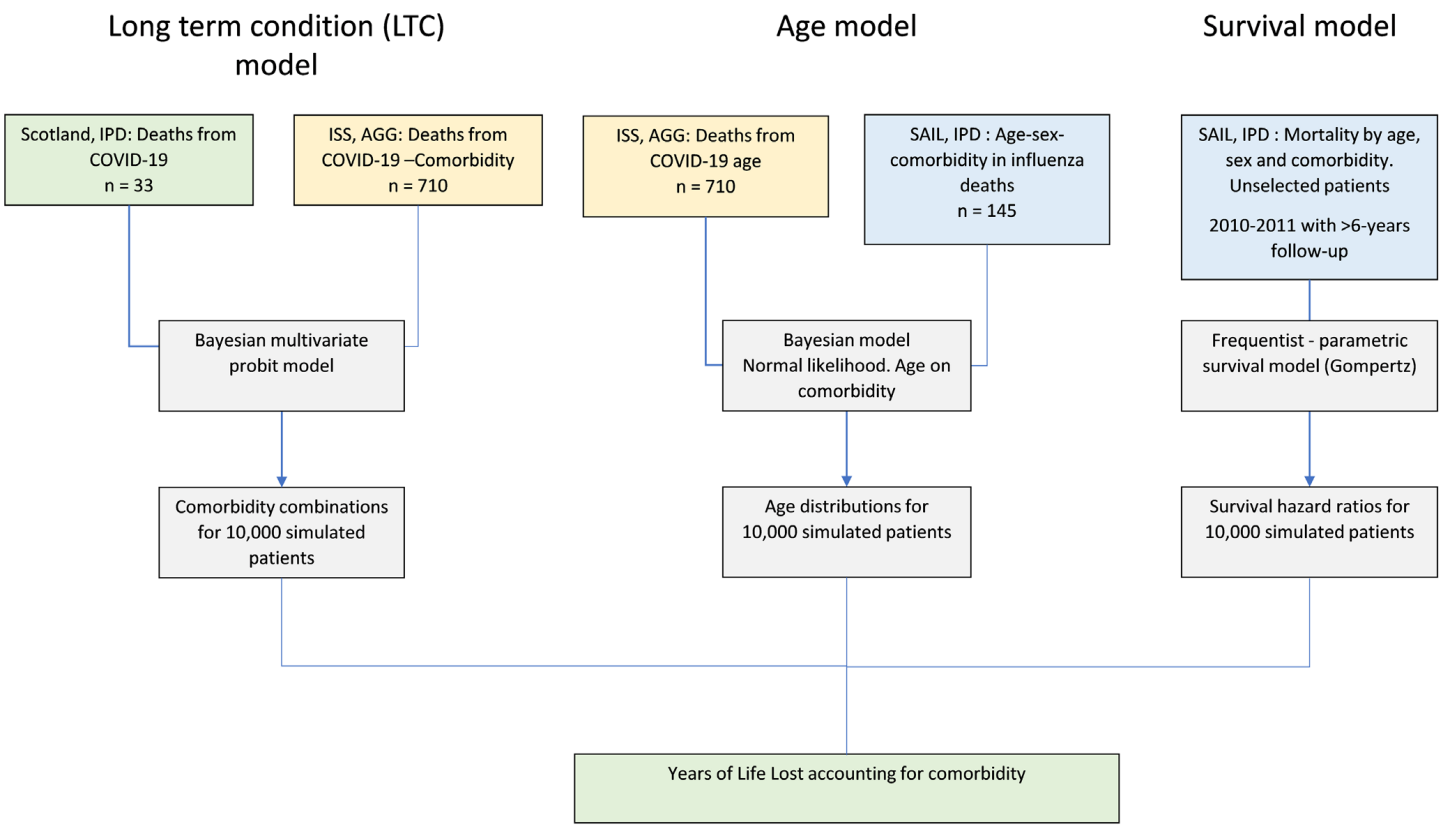
Overview of components of models. Green boxes indicate source of data/final outputs. Yellow boxes indicate Istituto Superiore di Sanità (ISS) data and blue boxes indicate Secure Anonymised Record Linkage (SAIL) data. White boxes indicate each model used to inform the final analysis. AGG - aggregate. IPD - individual level patient data.

The data sources used for each of these stages of modelling are summarised in Figure 1.

### Rapid review

To inform our estimates of number and type of LTCs, we first sought to identify the most detailed data available for underlying long-term conditions among people dying of COVID-19. We performed a rapid review to identify data on underlying conditions for people dying with COVID-19. We searched the WHO repository of COVID-19 studies on 24 ^th^ March 2020. To identify studies reporting data on LTCs among people who had died from Covid-19, we screened titles and abstracts of all epidemiological, clinical, case-series and review articles (n=1685). We identified and screened 77 potentially relevant full-text articles, of which four reported aggregate data on LTCs among people who had died of COVID-19. Three were small studies (32, 44, and 54 deaths, respectively) based in Wuhan, China ^5– 7^. However, the fourth was a comprehensive report from the Istituto Superiore di Sanità (ISS) (published each Tuesday and Wednesday) including data on 11 common LTCs (ischaemic heart disease, atrial fibrillation, heart failure, stroke, hypertension, diabetes, dementia, chronic obstructive pulmonary disease, active cancer in the past 5 years, chronic liver disease and chronic renal failure), as well as the number of patients who had 0, 1, 2 or ≥3 LTCs for 701 of the 6801 people who died with COVID-19 in Italy ^14^. In view of the smaller sizes of the Chinese studies, and the greater dissimilarity of these populations with the UK relative to the Italian data, we opted not to include these in the analysis. These data were used to construct a plausible scenario for the prevalence of combinations of LTCs among people who died from COVID-19 for the modelling presented here.

***Long-term condition prevalence and correlation models.*** This first stage of our modelling aimed to estimate the prevalence and correlation between specific LTCs among people dying with COVID-19.

We utilised aggregate data on COVID-19 deaths from the Istituto Superiore di Sanità in Italy. Since we were unable to obtain individual patient data for the Italian case-series of deaths from COVID-19, we had to infer the joint prevalence of LTCs from the summarised information available, i.e. the marginal distribution of multimorbidity counts (the row sums, or total number of diseases for each patient, wherein counts of ≥3 LTCs were collapsed into the single category of 3+) and the marginal distributions of LTC frequency (the columns sums, or the total number of patients with each LTC). To that end, we developed a Bayesian latent process model of disease prevalence and correlation and fitted it using Markov chain Monte Carlo (MCMC) to both elements in the published data. This analysis was applied jointly to the small number of deaths that had occurred in Scotland, primarily to aid convergence in Bayesian model fitting by providing some information about the correlation between LTCs ^15^. The Scottish subset of the data contained a partial record of known LTCs for individual patients, but the multimorbidity count per patient, as well as the marginal frequency of each LTC, were missing (hence, modelled as latent). Bayesian priors for the correlations between diseases were specified with a tendency to zero (shrinkage). Numerical investigations indicated little sensitivity of convergence to the strength of shrinkage, so we opted for weak shrinkage as a precautionary approach. This model gave us the full matrix of correlations between every combination of LTCs at the level of individuals, therefore providing us with a complete dependence structure of LTCs presented within the sample of COVID-19 mortalities. In order to propagate uncertainty through the analysis, from this fitted model (effective sample size of MCMC 410) we simulated 10,000 notionally “typical” patients, with plausible combinations of LTCs (under the combined Italian and Scottish data).

To test the sensitivity of our findings to the estimated correlations, we also estimated the YLL under two opposite extremes (i) that LTCs were independent and (ii) that LTCs were highly correlated. Unlike the Bayesian LTC mode, these sensitivity analyses did not use the information on the multimorbidity counts from the ISS report, but only the proportion of patients with each of the eleven comorbidities. For the “independent” scenario we created 11 vectors comprising 1s and 0s (respectively with and without the long term condition) corresponding in length to the number of patients. We then sampled from these vectors with replacement to obtain 10,000 simulated patients. For the “highly correlated” scenario we first sorted each vector, then combined them to form a 710x11 matrix, then sampled each row with replacement to obtain 10,000 simulated patients. This generated a dataset where individuals with one comorbidity which reduces life expectancy were more likely to have other comorbidities which reduce life expectancy (and vice versa).

***Age models.*** Next, we modelled the relationship between age and multimorbidity counts among people dying with COVID-19. We were unable to obtain direct estimates of the association between age and extent of multimorbidity among patients who had died from COVID-19. Therefore, we modelled two scenarios: independence between age and multimorbidity count (i.e. no correlation between age and multimorbidity count among people dying of COVID-19), and a positive association between age and multimorbidity count. To inform the latter, we examined data within the Secure Anonymised Information Linkage (SAIL) databank for 145 patients who had influenza recorded as the cause of death in their death certificate in 2011. SAIL is a repository of routinely collected healthcare data (including primary care, hospital episodes, and mortality data) from a representative sample covering approximately 70% of the population of Wales. While influenza is a different condition, these data were used for the sole purpose of estimating correlations between age and multimorbidity counts (conditioning on death), and did not inform the model in any other way. We found that for men, age increased by 4.7 years per unit increase in the number of LTCs until the count reached 6 after which there was no evidence of further increase. For women, the figure was 2.6. Therefore, we performed the modelling assuming that for COVID-19 the mean age increased by 5 years per unit increase in multimorbidity count across the range from 0 to 6 LTCs in men. To allow for some degree of uncertainty around this estimate by sampling from a normal distribution. We arbitrarily chose a standard deviation of 0.5. We estimated this similarly for women, but using a mean increase of age of 3 years per increase in multimorbidity count. We incorporated this information in a model fitted to the summary age data provided in the Italian case report. We obtained 10,000 samples from the posterior distribution for inclusion in the YLL calculations. SAIL analyses were approved by SAIL Information Governance Review Panel (Project 0830). Approval for the use of individual patient data in the analysis was given by the NHS Public Health Scotland Caldicott officer.

***Survival models.*** For patients aged 50 years or older at death, we estimated mortality according to age, sex and combinations of each LTC using the SAIL. From these data, we identified all participants aged over 49 years who were registered with a participating practice for the duration of 2011 (approximately 0.85 million people). This period was selected as electronic coding of diagnoses was well established, and it allowed >6 years of follow-up. Age and sex were extracted from primary care records. We also identified all LTCs for which we had information of COVID-19 deaths from Italy. LTCs were identified using a combination of primary care data (using Read diagnostic codes) and hospital episodes (using ICD-10 codes). Individuals were considered to have a LTC if they had a relevant diagnostic code entered prior to 31 ^st^ December 2011. Relevant codes were identified from the Charlson comorbidity index and the Elixhauser comorbidity index ^16, 17^, which had established algorithms for identification from ICD-10 codes ^18^, and have been adapted for using Read codes in primary care ^19^. Code lists are available in the supplementary material ^15^.

All-cause mortality was assessed by linkage to national mortality registers from 1 ^st^ January 2012 until August 2018 (last available data). Participants were censored if they de-registered from a participating SAIL practice. We used the flexsurv package in R (version 1.1.1) to fit a Gompertz model treating age as the timescale ^20^. We assessed the fit of this distribution graphically (supplementary material) ^15^. In models stratified by sex we included all the LTCs as main effects as well as age-LTC interactions that improved the model fit in terms of the Akaike information criterion. In sensitivity analyses we also included two-way (comorbidity-comorbidity) and three-way (comorbidity-comorbidity-age) interaction terms for the four comorbidities with the largest effect measure estimates (COPD, heart failure, liver failure and dementia) requiring 12 additional parameters. To propagate uncertainty from the survival models we obtained 10,000 samples of the coefficient estimates by sampling from a multivariate normal distribution corresponding to the coefficients and variance-covariance matrix from the regression models.

***Combination of comorbidity and mortality models.*** In the final analysis, we combined 10,000 samples from all three sources: LTC combination models, age models and survival models. We used the rate and shape parameters with the cumulative distribution function implemented in the flexsurv package to calculate the survival probabilities at 3-month intervals from aged 50 to 120 (to allow all curves to descend to zero). From these times and survival probabilities we estimated the mean survival, or life expectancy.

Bayesian models were written in the JAGS language ^21^ and implemented using runjags for R (version 2.0.4) ^22^, survival models were fit using the flexsurv package in R (version 1.1.1) ^20^, and for the final analysis the model-outputs were also combined in R (version 3.6.1). The 95% uncertainty intervals were obtained using empirical bootstrapping, with the number of samples in the mean equal to the effective sample size from the LTC correlation model. All code, data (except individual-level data for Scotland), intermediate outputs and diagnostic plots are provided on GitHub ( https://github.com/dmcalli2/covid19_yll_final) ^15^.

## Results

### WHO life tables

The proportion of men and women in 10-year age-bands was reported for the 6801 deaths included in the ISS case report. On applying the proportion in each age-band to the WHO Global Burden of Disease 2010 life tables for men, we found that the YLL was 14.4 per person using the whole cohort and 14 after excluding those aged under 50. For women, comparable figures were 12.2 and 11.8 years, respectively. In sensitivity analyses using alternative life tables, life expectancy was lower (particularly for men), however the estimates YLL remained above 10 years for both men and women, regardless of life table used (detailed results shown in https://github.com/dmcalli2/covid19_yll_final/blob/master/Scripts/Addendum.md).

### Comorbidity models

For 710 patients who had died with COVID-19 for whom information on LTCs was presented in the ISS report ^14^, the proportion with each LTC was as follows:- ischaemic heart disease 27.8%, atrial fibrillation 23.7%, heart failure 17.1%, stroke 11.3%, hypertension 73%, diabetes 31.3%, dementia 14.5%, chronic obstructive pulmonary disease 16.7%, active cancer in the past 5 years 17.3%, chronic liver disease 4.1%, chronic renal failure 22.2%. The ISS report also presented the proportion of patients who died with each of the following multimorbidity counts: 0 (2.1%), 1 (21.3%), 2 (25.9%) and ≥3 (50.7%). Using these data, alongside individual-level patient data for a small number of patients from Scotland to aid with model fitting, we were able to simulate a set of realistic notional patients with specific combination of LTCs. The correlations between every pair of LTCs are shown in the appendix and the full posterior distributions from the modelling are available at GitHub ( https://github.com/dmcalli2/covid19_yll_final) ^15^.

### Age models

**Figure 2.**
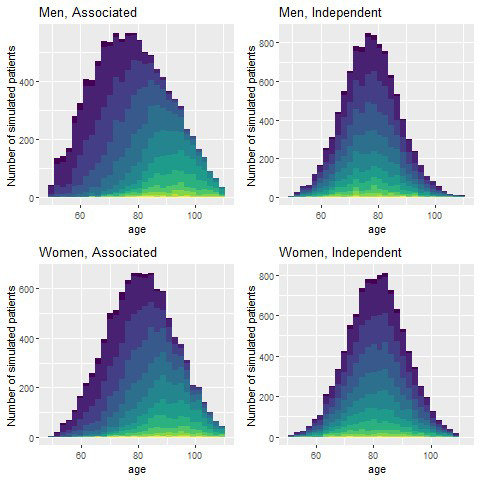
Modelled distribution of age in ISS population, assuming age is associated with comorbidity counts, and assuming age and comorbidity are independent. Coloured bars indicate the comorbidity count from zero (dark/blue) to 11 (light/yellow).

Based on the proportions reported for each age-band, for men the mean age for the ISS deaths was 77.9 years when people aged less than 50 were excluded and 77.4 years overall. For women the figure was 81.1 for both. The models we fit to these data to smooth out the distribution and to make it easier to accommodate different scenarios for the association between age and multimorbidity counts comorbidity are shown in Figure 2; the distribution of age and multimorbidity counts for men and women are shown under the assumption that these are independent, and under the assumption that multimorbidity is associated with age.

### Survival models

**Figure 3.**
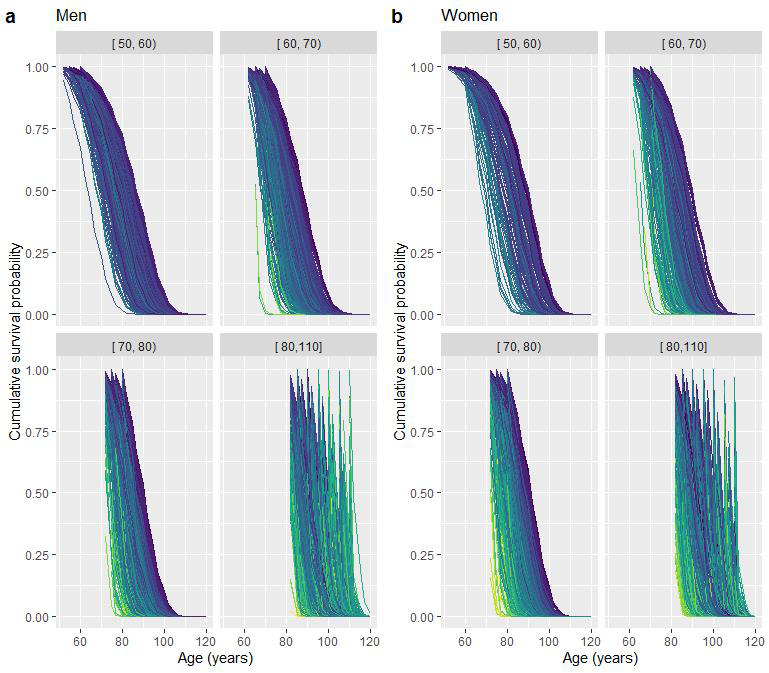
Survival curves for all-cause mortality. Figures are paneled by age and sex. Individual lines represent survival curves for a single simulated patients with a given set of LTCs. From light to dark (yellow to blue) they show decreasing multimorbidity counts (11 to 0). There are 10, 000 lines, one for each notional patient. Lines run from the age at which each simulated patient died (survival probability = 1) to when they would have died under the model (survival probability = 0). Patients with the same age and total multimorbidity count will have a different survival curve if they have a different set of 11 LTCs.

The coefficients for the survival models are shown in the supplementary appendix. Briefly, all LTCs other than hypertension were associated with increased mortality (in a model including 10 other LTCs), and for each LTC the association with mortality was attenuated as the baseline age increased. Figure 3 shows the survival curves applied to different age and combinations of LTCs, stratified by age-band and multimorbidity count. This figure shows how these associations and age relate to survival across the age range from 50 to 110 years old.

### Years of life lost

For men the average YLL on adjusting for number and type of LTC as well as age was 11.6(10.9–12.4). For women this value was 9.4(8.7–10). The results were similar under the different assumptions for the age-multimorbidity association and in both sensitivity analyses, whether assuming strongly correlated or independent LTCs ( Table 1). For comparison, the YLL based on age alone using the WHO tables was 14.0 and 11.8 for men and women, respectively.

**Table 1.** Years of life lost (YLL) and 95% credible intervals under different modelling assumptions.

LTC-LTC correlation	Age- multimorbidity correlation	Men	Women
Modelled	Associated	11.6 (10.9-12.4)	9.4 (8.7-10)
Modelled	Independent	11.1 (10.4-11.7)	9.2 (8.6-9.8)
Independent	Associated	12 (11.2-12.9)	9.8 (9.2-10.5)
Independent	Independent	11.5 (10.9-12.1)	9.6 (9.1-10.2)
Highly correlated	Associated	13.5 (12.5-14.4)	10.9 (10.1-11.8)
Highly correlated	Independent	12.8 (12.1-13.6)	10.7 (10-11.5)

**Figure 4.**
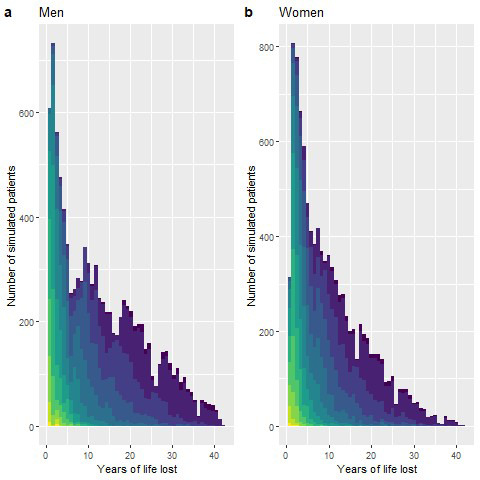
YLL by sex. Coloured bars indicate the multimorbidity count from zero (dark/blue) to 11 (light/yellow).

Across the simulated patients there was substantial variation in YLL adjusted for multimorbidity count ( Figure 4).

**Figure 5.**
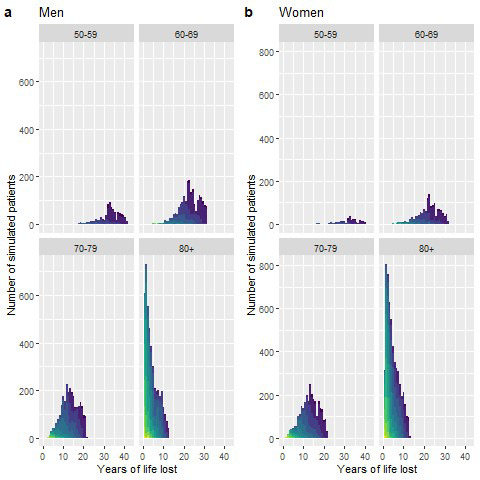
YLL stratified by sex, age and multimorbidity count. Coloured bars indicate the multimorbidity count from zero (dark/blue) to 11 (light/yellow).

On stratifying the YLL estimates by sex, age and multimorbidity count (for the simulated patients) there were clear differences ( Figure 5, Table 2) with the YLL ranging from around 2-years per person in men or women aged 80 with large numbers of LTCs, to around 35 years in younger people without any LTCs ( Table 2). For most age-bands and most multimorbidity counts the YLL per person remained above 5. In sensitivity analyses including the survival models with additional comorbidity-comorbidity and comorbidity-comorbidity-age interaction terms, (despite these models having a better fit based on AIC) than the model presented here, the YLL only changed minimally from that seen in the main analysis. This was true overall YLL for each sex (13.1, 95% CI 12.2–14.0 and 10.5; 95% CI 9.7–11.3 for men and women respectively) and on additionally stratifying on age and multimorbidity count (as shown in Table 2). For the latter comparison, the largest difference – 0.7 YLL – was seen in women aged 50–59 with six comorbidities. For most age-comorbidity bands the YLL was the same, to one decimal place, under both survival models.

**Table 2.** Mean years of life lost, accounting for type of long-term conditions *, by age-band, sex and multimorbidity count.

	Men				Women			
Multimorbidity count	50-59	60-69	70-79	80+	50-59	60-69	70-79	80+
0	35.37	25.76	16.83	7.29	33.59	26.40	17.00	6.85
1	34.99	25.42	16.73	6.69	35.12	25.51	16.62	6.99
2	30.04	22.36	14.58	5.78	28.76	21.41	14.37	6.09
3	26.49	19.01	12.35	5.14	25.31	18.26	11.94	5.31
4	22.00	15.93	10.64	4.36	20.27	15.27	10.07	4.46
5	18.27	13.79	9.07	3.60	16.63	12.70	8.28	3.84
6	14.63	11.09	7.26	-	11.67	9.61	6.57	3.27
7	11.32	9.44	6.08	2.56	9.82	7.67	5.05	2.76
8	7.68	6.97	4.56	2.03	6.62	5.48	3.88	2.33
9	-	5.81	3.84	1.64	-	3.64	2.80	1.60
10	-	-	4.14	-	-	-	2.71	

*Estimates are based on life-expectancy calculates for specific types and combinations of LTCs, which are then aggregated across LTC counts.

## Discussion

### Summary of main findings

Using published data on people who have died from COVID-19 and survival models based on age and multimorbidity count in a general population in the United Kingdom, we estimated the burden (years life lost) from COVID-19 related mortality. We make a number of important observations. First, using the WHO GBD 2010 life tables as the reference ^13^, the estimated YLL was over a decade for COVID-19 deaths with 14 YLL in men and 12 in women. As such, mortality from COVID-19 represents a substantial burden to individuals and comparable to high burden LTCs such as ischaemic heart disease and chronic obstructive pulmonary disease. Second, YLL estimated from models using the prevalence of underlying LTCs based on patients dying from COVID-19 in Italy and age-, sex- and multimorbidity count-specific survival models in the UK did not drastically impact the YLL. Across both men and women, the number of YLL dropped to 11.6 and 9.4 years respectively. Third, across most age and multimorbidity count strata the estimated YLL per person remained substantial and generally above 5 years. This means that even after accounting for multimorbidity count, most individuals lost considerably more than the “1–2 years” suggested by some commentators ^23^ perhaps ^24, 25^ reflecting the high prevalence of multimorbidity in this population, especially in those over the age of 50 years ^26, 27^. Finally, whilst the YLL remained high across most age- and multimorbidity count strata, the presence of multimorbidity did indeed influence the magnitude of the YLL. For example, in the elderly, over the age of 80, the estimated YLL in people with no LTCs was 7 years falling to less than two years with an increasing multimorbidity count.

YLL is a widely used metric to compare the relative impact of different causes of death and is used to guide policy-making and health service delivery and to prioritise interventions aimed at preventing deaths ^28^. Using UK reports for approximate comparisons, the YLL in England and Wales for other conditions ranged, per capita from 8.2 for chronic obstructive pulmonary disease, 11.6 for coronary heart disease, 13.1 for pneumonia, and 21.6 for asthma ^29^. Therefore, against these benchmarks, mortality from COVID-19 represents a substantial burden to individuals. It should be noted, however, that YLL for an emergent infection such as COVID-19, particularly in a pandemic, will be sensitive to the specific circumstances of the virus spreading, mitigation strategies, and potential future treatment or vaccines. These estimates, therefore, relate to the specific conditions at the time of modelling and will need to be updated particularly as vaccination or other strategies alter susceptibility or severity of infection. It is important to note, however, that it would be a misuse of any such modelling if it were used to criticise decision-making undertaken at the time.

The estimated YLL can vary substantially depending on the reference population chosen and the age distribution among those who die. Moreover, where attempts are made to account for underlying conditions in those who died, the accuracy will depend on the quality and completeness of data both for those deaths, and in the reference population used to obtain estimates of survival according to those underlying conditions. Nonetheless, although imperfect, we would argue that public health agencies should present estimates of YLL for COVID-19, alongside the more usual counts of deaths. We have already seen that if agencies do not do so, commentators can and will fill this vacuum, sometimes making substantial errors such as using life expectancy at birth to make inferences about the years of life lost by someone who has already lived into later life and thereby considerably underestimating the impact of the disease on individuals ^23^. In additional to reporting YLL, metrics such as excess deaths and quality-adjusted life years are important to fully contextualise the loss of life seen in the pandemic.

It should be noted that these estimates were made early in the pandemic and could not account for specific patterns and events which emerged within the UK. For example, these analyses were performed before the impact of COVID-19 in care homes in the UK became apparent. SAIL contains data on all participants registered with a GP (and so would include care-home residents), however our estimates of life expectancy do not distinguish between people who live in care-homes and those who do not. As such our analyses would not reflect the YLL at a population level where care-homes are disproportionately impacted. Our estimates, given the data sources which were available at the time, are more likely to reflect the YLL of COVID-19 deaths among hospitalised patients.

Finally, our estimates of YLL only attempt to quantify the direct effects of COVID-19. Indirect impacts on mortality (e.g. through pressure on healthcare services of unintended consequences of lockdown measures) should also be considered, and are not captures by our YLL calculation.

### Strengths and limitations

Our analysis is novel in that it adjusts YLL for the number and type of underlying LTCs. This is important as people with underlying multimorbidity are recognised to be more vulnerable to COVID-19. However, although we had data for eleven common and important LTCs, we did not have markers of underlying disease severity among those who died. Severity of the underlying LTC has considerable impact on life expectancy ^30^. Moreover, we had no data for rarer severe LTCs, which may nonetheless be common among those who die from COVID-19 at younger ages. As such, the attenuation of YLL following adjustment for LTCs may be an underestimate. However, we think that this effect is unlikely to be substantial enough to reduce YLL to the orders of magnitude suggested by some commentators. Indeed, on stratifying by age and multimorbidity counts, we rarely found average YLLs of below three. Also, we were not able to adjust our estimates for other factors and exposures (such as socioeconomic status, occupation, smoking, health behaviours) which would have given a more accurate representation of life-expectancy in the absence of COVID-19.

Socioeconomic status is a particularly pertinent issue, as it may influence not only outcomes from infection (e.g. through multimorbidity and other risk factors) but also the likelihood of exposure (e.g. higher proportions of occupations for which home-working was not feasible). Since socio-economic status also predicts mortality there is a possibility of residual confounding due to the lack of data on socioeconomic status available for our models. To prevent mean inflation through rare deaths in younger people, who only modelled deaths in people over 50 years, however deaths among younger people may influence estimates YLL.

We did not have access to large quantities of individual-level data with which to estimate the prevalence of different combinations of LTCs. Therefore, we fitted a complex model (which was methodologically innovative and will be the subject of a separate publication) to estimate the joint probabilities, using the overall (marginal) estimates of each LTC, and the overall multimorbidity counts alongside a small amount of individual-level data from Scotland to help with model fitting. This model (i) represents the best estimate for the joint probabilities given the available data and importantly, (ii) the results for overall YLL remained substantially similar in widely different sensitivity analyses assuming either that LTCs are highly correlated among people dying from COVID-19 or that they are entirely independent.

Finally, given the emergent nature of the coronavirus pandemic, this study was conducted rapidly and under pressure of time. We chose the best data for age, sex and prevalence of LTCs that was available to us at the time of our modelling, but better-quality individual-level data specific to individual countries will yield substantially more reliable estimates. We would suggest that each public health agency should produce country-specific estimates, using the same LTC definitions in those who died as in the reference population and ideally to an agreed international protocol. Our study has used complex state-of-the-art statistical modelling and inference techniques, which rely on expensive computer simulations. We have also provided all our data (except individual-level data form the Scottish population, for which we provide a simulated substitute dataset) and code to allow others to check our modelling and correct any errors ^15^.

Our model, due to limited data available at the time, combined data on Covid-19 deaths and life expectancy data from different countries and contexts. While this synthesis of data sources allowed an estimation to be generated at an early stage, it limits the generalisability to specific contexts. Summaries of YLL relating to a specific country or context should ideally use data (both life-expectancy and Covid-19 related) from that context. A comparison of such estimates (based on individual-level and country specific data) with our approach (modelling aggregate- and individual-level data from multiple sources early in the pandemic) would be important to test the utility of this approach for future pandemics.

Despite these limitations, our findings do indicate that adjusting for number and type of LTCs does not substantially reduce the estimated YLL compared to the standard approach. Our analysis does not, however, offer a definitive estimation of YLL across all contexts, nor does it necessarily fully adjust for underlying health status. For example, further work based in Scotland has illustrated that the life expectancy in care-home residents, and therefore the estimated YLL, is substantially different from the general population ^31^. This is important given the large proportion of COVID-19 deaths that have occurred in care homes ^32, 33^. Additionally, it indicates that additional factors are likely to influence underlying health status, life expectancy, likelihood of dying from Covid-19, and by extension YLL. These factors are not fully represented by the presence or absence of specific LTCs. Some of these factors are likely to be challenging to estimate from routine data alone, and producing YLL estimates which account for these factors should be an area of future investigation.

## Conclusion

Among patients dying of COVID-19, there appears to be a considerable burden in terms of years of life lost, commensurate with diseases such as coronary heart disease or pneumonia. While media coverage of the pandemic has focused heavily on COVID-19 affecting people with ‘underlying health conditions’, and while the number and type of LTCs certainly influence the life expectancy and YLL for individuals, adjustment for number and type of LTCs only modestly reduces the estimated YLL due to COVID-19 compared to estimates based only on age and sex. Public health agencies and governments should report on YLL, ideally adjusting for the presence of underlying LTCs, to allow the public and policy-makers to better understand the burden of this disease.

## Data availability

All code, data (except individual-level data for Scotland), intermediate outputs and diagnostic plots are provided on GitHub: https://github.com/dmcalli2/covid19_yll_final.

### Source data

Zenodo: Data and Code to support COVID-19 - exploring the implications of long-term condition type and extent of multimorbidity on years of life lost: a modelling study. https://doi.org/10.5281/zenodo.3751561 ^34^.

This project contains the source data used in performing this modelling study (except individual-level data for Scotland), which are also available via GitHub ( https://github.com/dmcalli2/covid19_yll_final/tree/master/Data).

Individual-level data for Scotland are accessible via application to the electronic Data Research and Innovation Service (eDRIS) and the Public Benefit and Privacy Panel (PBPP) ( https://www.isdscotland.org/Products-and-Services/EDRIS/). Individual-level data for Wales are available via application to the Secure Anonymised Information Linkage (SAIL) at https://saildatabank.com/. For both eDRIS and SAIL, individuals are required to complete information governance training, be affiliated with an appropriate organisation (e.g. a university, healthcare organisation, etc.) complete an application form, and the analysis must be performed to support research conducted in the public interest.

### Extended data

Zenodo: Data to support COVID-19 - exploring the implications of long-term condition type and extent of multimorbidity on years of life lost: a modelling study. http://doi.org/10.5281/zenodo.3751561 ^34^.

This project contains the archived scripts used during this modelling study, which are also available via GitHub ( https://github.com/dmcalli2/covid19_yll_final/tree/master/Scripts).

Data are available under the terms of the Creative Commons Attribution 4.0 International license (CC-BY 4.0).
