# Supplementary material for: COVID-19 – exploring the implications of long-term condition type and extent of multimorbidity on years of life lost: a modelling study
Source: Wellcome Open Res. 2021 Mar 1;5:75. Originally published 2020 Apr 23. [Version 3] doi: 10.12688/wellcomeopenres.15849.3 (PMC7927210; doi:10.12688/wellcomeopenres.15849.3)
Supplement: Peer review history

# Peer review: Reviewer response for version 3

**DOI:** 10.21956/wellcomeopenres.18386.r42884

**Published:** 2021-03-29

**Authors:** Mei Sum Chan

**Affiliations:** 1 University of Oxford, Oxford, UK; 2 University College London, London, UK

Thank you for your responses to my earlier comments, and for the changes to the manuscript. The manuscript and the underlying analysis have been much improved by these changes. The rationale for investigating YLL by multimorbidity is now much clearer and the manuscript and supplementary material are much better organised. I am reassured of the validity of the results, now that the model converges and the estimates have not changed substantially. To support this study, the separate methodological investigation that the authors mentioned would still be useful in providing further validation and technical details, as well as investigating the trade-offs in model constraints vs convergence and the effects of using alternative data and model structures.

 I am happy to approve this version of the manuscript, but recommend that the manuscript be further improved through consideration of the following:

 Since ‘long-term condition type’ still appears in the title, and the modelling approach still differentiates between specific LTC combinations, it would be more consistent to either change the title or report the intermediate results by LTC type, for example in the supplementary material.

1. Please spell out the first mention of ‘YLL’ in the abstract.
2. The new text in the introduction ‘This manuscript presents a revised version of an analysis initially conducted in March 2020’ appears to give context for the choice of method (sparse data), but the initial analysis that is alluded to is not described in the introduction. A clearer justification for the study design in the introduction would improve readability.
3. Regarding my earlier comments on the mixture of selected and unselected populations used across the models (illustrated in Figure 1), the authors have added further explanation of constraints on data availability, which was useful. However, the use of this mixture is not just attributable to the pandemic stage at the time of analysis (as the authors mentioned), but also the choice of model structure.

Is the work clearly and accurately presented and does it cite the current literature?

Partly

If applicable, is the statistical analysis and its interpretation appropriate?

I cannot comment. A qualified statistician is required.

Are all the source data underlying the results available to ensure full reproducibility?

Yes

Is the study design appropriate and is the work technically sound?

Partly

Are the conclusions drawn adequately supported by the results?

Partly

Are sufficient details of methods and analysis provided to allow replication by others?

Yes

Reviewer Expertise:

Epidemiology, medical statistics, gerontology.

I confirm that I have read this submission and believe that I have an appropriate level of expertise to confirm that it is of an acceptable scientific standard.

# Peer review: Reviewer response for version 3

**DOI:** 10.21956/wellcomeopenres.18386.r42885

**Published:** 2021-03-02

**Authors:** Jonathan Stokes

**Affiliations:** 1 Division of Population Health, Health Services Research & Primary Care, University of Manchester, Manchester, UK

The authors have addressed all of my remaining comments, the article is now fairly balanced across the abstract/main text. Congratulations on a very interesting paper!

Is the work clearly and accurately presented and does it cite the current literature?

Partly

If applicable, is the statistical analysis and its interpretation appropriate?

I cannot comment. A qualified statistician is required.

Are all the source data underlying the results available to ensure full reproducibility?

Partly

Is the study design appropriate and is the work technically sound?

Partly

Are the conclusions drawn adequately supported by the results?

Partly

Are sufficient details of methods and analysis provided to allow replication by others?

Partly

Reviewer Expertise:

Research Fellow in Health Economics, PhD, and Master's of Public Health. Expertise in evaluating new models of care for multimorbid patients.

I confirm that I have read this submission and believe that I have an appropriate level of expertise to confirm that it is of an acceptable scientific standard.

# Peer review: Reviewer response for version 3

**DOI:** 10.21956/wellcomeopenres.18386.r42883

**Published:** 2021-03-02

**Authors:** Ramon Martinez-Piedra

**Affiliations:** 1 Department of Non-Communicable Diseases and Mental Health, Pan American Health Organization, Washington, DC, USA

I'm glad of this new version of the article, and the responses provided by authors. No comments to add.

Is the work clearly and accurately presented and does it cite the current literature?

Yes

If applicable, is the statistical analysis and its interpretation appropriate?

I cannot comment. A qualified statistician is required.

Are all the source data underlying the results available to ensure full reproducibility?

Yes

Is the study design appropriate and is the work technically sound?

Yes

Are the conclusions drawn adequately supported by the results?

Yes

Are sufficient details of methods and analysis provided to allow replication by others?

Yes

Reviewer Expertise:

Epidemiology, public health, health metrics, data anlysis.

I confirm that I have read this submission and believe that I have an appropriate level of expertise to confirm that it is of an acceptable scientific standard.

# Peer review: Reviewer response for version 2

**DOI:** 10.21956/wellcomeopenres.18306.r42514

**Published:** 2021-02-16

**Authors:** Jonathan Stokes

**Affiliations:** 1 Division of Population Health, Health Services Research & Primary Care, University of Manchester, Manchester, UK

Dear Authors,

 Thank you for taking the time to respond to my previous comments. The additional limitations and clarifications have already improved the manuscript significantly. I do, however, still have comments which I do not feel have been adequately addressed and require amendment to ensure scientific rigour. Additionally, a few of my previous comments appear to have been mis-interpreted (apologies for any lack of clarity), so I provide a few clarifications at the end.

**Comment requiring additional input**:

 Your main clarification is: “Our analysis was therefore an attempt to model, at a very early stage in the pandemic and with limited and incomplete data, the impact of COVID-19 on years of life lost (YLL)… Our analytic approach was specifically designed and developed to estimate YLL in spite of the sparse data available at the time. It was highly novel in that it used aggregated data on comorbidity to derive estimates of combinations of comorbidity. We did not, and do not, suggest that ours should be the definitive estimate of YLL.”

 I appreciate this, effectively you argue it is a methods piece then (almost), providing a framework that can potentially “be useful for early-stage estimation in future pandemics”. I agree, this is novel and interesting.

 However, while the manuscript now captures these limitations fairly well, the abstract does not, whatsoever. This is particularly important since this is all most people are likely to read and it could be extremely misleading. It is currently framed as an estimation of, and implies policy conclusions around, the YLL figure(s). I would suggest, as a minimum, amending the abstract to make the data issues clear and up-front and that the estimated YLL is potentially flawed for this reason.

 If it is primarily a methods paper though, i.e. with any scope for usefulness for policymaking at an early stage of future pandemics as you suggest, I would also expect validation of the methods. The adage “all models are wrong, but some are useful” comes to mind. The method only has scientific/policy value if the conclusions the model gives reflect reality. It is not useful if it would give a completely different answer given better data, and so would imply different policy decisions to what would have been enacted (the model needs to effectively mimic the results given ‘gold standard’ data). Having used “sparse and aggregate data” it is currently still not obvious without this validation whether the model is actually useful in a policy/scientific sense or whether waiting for good quality data is a necessary input to accurately predict YLL accounting for multimorbidity. Ideally, as in my previous review, I would still like to see the method validated in some way, e.g. with more robust updated data from a coherent setting and not across multiple countries, and the manuscript presented as a methods piece. (As above, however, I do see that the manuscript now better reflects the limitations of the study, so deemphasising the policy significance of the presented YLL figure (particularly in the abstract), might be enough for this paper if you plan to do this validation separately. If this is the case though, you should probably add an additional limitation to this version that the method requires validation prior to recommending use in future pandemics.)

**Clarifications to previous comments**:

- “Author response. Thank you for these comments. Taking first the issue of using data from patients who have died to estimate YLL…it is necessary to use data from people who have died…” - The issue is not taking ‘people who have died’, of course data on deaths is required to estimate YLL (thank you for pointing this out). The issue is, as I alluded to in other comments, if all (or half, more reasonably, even) the people who died were in a care home, as an example, the life expectancy of this selected group is very different from a general population of the same age (median, around 20 months has been estimated, although data is not great - https://www.pssru.ac.uk/pub/3211.pdf). You need to be able to say something about observables (co-variates) of the population of people in your death dataset compared to observable of deaths (/life expectancy) in the general population (especially when the deaths and the life expectancy are from completely different countries/contexts). That is the potential selection effect I mentioned, i.e. it is likely hiding a lot of other confounding. From your response, though, it seems like you are trying to demonstrate what is possible to estimate with flawed data at the start of a pandemic, which is fine. In which case, I would expect this selection effect to be picked up in the limitations (like you have now incorporated in response to my other comments).
- “The standard approach to YLL typically uses just age and sex. We develop this further to include comorbidity, to address the specific point raised in the context of COVID-19, that because of common comorbidities most people who died with COVID-19 would have died soon in any case”. Yes, but adding ‘comorbidity’ in this way does not pick up the severity (proxied by care home/deprivation and other observables I mentioned). Sex and age are largely more straight-forward data points, but co-morbidity measurement is not (there is a lot of literature on this).
- “By definition, YLL cannot be observed, neither by hospital data, nor by controlled experiment…”. Again, thank you for pointing this out. But it can, of course, be estimated based on (either older or more updated) data, so it can be ‘observed’ in that sense. What I was getting at with this comment was similar to the above, if around half of the excess deaths are care home residents in most of Europe, and severe co-morbidity is frequently observed, then the YLL you estimate does not intuitively ‘fit’ with these additional observations from the existing literature (it might! But, it needs more work to validate first, I think. As you say, the YLL likely reflects a sub-population of non-care home/without severe conditions – potentially, the YLL in a system, Italy, having suffered long-term austerity and where the health system is overwhelmed). This is where I was getting at the need for some sort of validation in terms of latterly available (superior) data.
- “We would also note that none (to our knowledge) have attempted to model the observed events in care homes”. - Comas-Herrera *et al. *~42% recorded deaths on average, higher (around half) in earlier parts of epidemic ^1^. Reference 2[ref-] - excess care home mortality in England, ~55% of PHE estimated excess mortality in general population over same period.
- “life expectancy at birth. Once an individual is known to have survived to an older age, their life expectancy is much higher than it is at their birth”. Yes, but on average in population, not for most deprived/severely multimorbid/care homes, for instance.
- “Rather we calculate years of life lost for a given set of observed deaths. Since it is not possible to observe years of life lost we are not able to present an observed versus expected comparison”. Yes, but there is now better data on this, even from the same period you analyse. How does it compare to the updated set(s) of ‘observed deaths’?
- “To summarise, we think that this YLL can reasonably be generalised to high-income countries…”. Why is that reasonable? The Covid death profile in UK/Germany/Sweden/Denmark/etc. is completely different, isn’t it? Dependent on some combination of context-specific pre-existing conditions (e.g. ‘stock’ of health and risk factors in the population/resilience and capacity of services to respond) and reactive policy measures, surely?
- “However, since ten years is already rather a long period, we think that the former is the more pertinent for policy-making”. The more pertinent for policy-making should be the “true” value, surely (i.e. best available estimate given available up-to-date data). Would need to further demonstrate this with validation checks.
- “This conclusion has been built-upon via subsequent work by other[DM2]  researchers: [REFs]…” As far as I can see, none of these references estimate YLL accounting for multimorbidity or other relevant factors, though, so do not directly support the findings.
- “New data may show that there are other reasons (e.g., high levels of frailty) why the YLL is, after all, considerably lower for deaths from COVID-19 than the age, sex and comorbidity specific estimates suggest”. Personally, I would interpret the care homes literature/all of the literature on individual-level risk factors associated with mortality as already suggesting this. But, again, it’s an empirical question, would be great to see.

Is the work clearly and accurately presented and does it cite the current literature?

Partly

If applicable, is the statistical analysis and its interpretation appropriate?

I cannot comment. A qualified statistician is required.

Are all the source data underlying the results available to ensure full reproducibility?

Partly

Is the study design appropriate and is the work technically sound?

Partly

Are the conclusions drawn adequately supported by the results?

Partly

Are sufficient details of methods and analysis provided to allow replication by others?

Partly

Reviewer Expertise:

Research Fellow in Health Economics, PhD, and Master's of Public Health. Expertise in evaluating new models of care for multimorbid patients.

I confirm that I have read this submission and believe that I have an appropriate level of expertise to state that I do not consider it to be of an acceptable scientific standard, for reasons outlined above.

## References

[1] : Mortality associated with COVID-19 in care homes: international evidence. *Article in LTCcovid.org, International Long-Term Care Policy Network, CPEC-LSE*.2020;

[2] : Excess mortality for care home residents during the first 23 weeks of the COVID-19 pandemic in England: a national cohort study. *medRxiv*.2020; 10.1101/2020.11.11.20229815 10.1101/2020.11.11.20229815 PMC793276133663498

# Response

**Published:** 2021-02-19

**Authors:** Peter Hanlon

**Affiliations:** University of Glasgow, UK

Thank you for your comments that the previous revision has significantly improved the manuscript. Thank you also for your further comments and clarifications which we respond to below. In short, we agree with the need to revise the abstract to reflect the clarifications and limitation highlighted within the main text. We also agree with the need to interpret the policy-relevance of the findings in the context of their limitations and have revised the discussion with this in mind. Our response to the specific points raised are detailed below along with all changes to the abstract and manuscript text.

**Reviewer comment:**

*Your main clarification is: “Our analysis was therefore an attempt to model, at a very early stage in the pandemic and with limited and incomplete data, the impact of COVID-19 on years of life lost (YLL)… Our analytic approach was specifically designed and developed to estimate YLL in spite of the sparse data available at the time. It was highly novel in that it used aggregated data on comorbidity to derive estimates of combinations of comorbidity. We did not, and do not, suggest that ours should be the definitive estimate of YLL.”*

*I appreciate this, effectively you argue it is a methods piece then (almost), providing a framework that can potentially “be useful for early-stage estimation in future pandemics”. I agree, this is novel and interesting.*

*However, while the manuscript now captures these limitations fairly well, the abstract does not, whatsoever. This is particularly important since this is all most people are likely to read and it could be extremely misleading. It is currently framed as an estimation of, and implies policy conclusions around, the YLL figure(s). I would suggest, as a minimum, amending the abstract to make the data issues clear and up-front and that the estimated YLL is potentially flawed for this reason.*

**Author response**

 Thank you for this comment, and for highlighting that the abstract requires updating to reflect the clarifications and limitations highlighted in the text. We have added the following to the background section of the abstract to highlight both the limitations of the data sources on which the findings are based:

 “We aim to estimate YLL attributable to COVID-19, before and after adjustment for number/type of LTCs, using the limited data available early in the pandemic.”

 And changes the final sentence of the conclusion to emphasise the points raised (particularly around residual confounding, and the lack of other markers of underlying health status):

 “More comprehensive and standardised collection of data (including LTC type, severity, and potential confounders such as socioeconomic-deprivation and care-home status) is needed to optimise YLL estimates for specific populations, and to understand the global burden of COVID-19, and guide policy-making and interventions.”

**Reviewer comment**

*If it is primarily a methods paper though, i.e. with any scope for usefulness for policymaking at an early stage of future pandemics as you suggest, I would also expect validation of the methods. The adage “all models are wrong, but some are useful” comes to mind. The method only has scientific/policy value if the conclusions the model gives reflect reality. It is not useful if it would give a completely different answer given better data, and so would imply different policy decisions to what would have been enacted (the model needs to effectively mimic the results given ‘gold standard’ data). Having used “sparse and aggregate data” it is currently still not obvious without this validation whether the model is actually useful in a policy/scientific sense or whether waiting for good quality data is a necessary input to accurately predict YLL accounting for multimorbidity. Ideally, as in my previous review, I would still like to see the method validated in some way, e.g. with more robust updated data from a coherent setting and not across multiple countries, and the manuscript presented as a methods piece. (As above, however, I do see that the manuscript now better reflects the limitations of the study, so deemphasising the policy significance of the presented YLL figure (particularly in the abstract), might be enough for this paper if you plan to do this validation separately. If this is the case though, you should probably add an additional limitation to this version that the method requires validation prior to recommending use in future pandemics.)*

**Author response**

 Thank you for these comments. We agree with the reviewer’s point about the limitations of this model to guide (particularly current) policy decisions or in the context of a future pandemic. It is also entirely fair that the abstract requires updating for this revised version. Changes to the abstract as a whole are detailed in the response to the comment above. We have added the following text to the conclusion of the abstract to emphasise the limitations of our model:

 “More comprehensive and standardised collection of data (including LTC type, severity, and potential confounders such as socioeconomic-deprivation and care-home status) is needed to optimise YLL estimates for specific populations, and to understand the global burden of COVID-19, and guide policy-making and interventions.”

 We would maintain that further work to estimate YLL based on updated or more detailed data that may now be available is beyond the scope of this manuscript, and will be better explored in separate studies focused on specific populations and policy contexts.

 We agree with the need for data from ‘a coherent setting’ – as you point out – and have added the following to the limitations:

 “Our model, due to limited data available at the time, combined data on Covid-19 deaths and life expectancy data from different countries and contexts. While this synthesis of data sources allowed an estimation to be generated at an early stage, it limits the generalisability to specific contexts. Summaries of YLL relating to a specific country or context should ideally use data (both life-expectancy and Covid-19 related) from that context. A comparison of such estimates (based on individual-level and country specific data) with our approach (modelling aggregate- and individual-level data from multiple sources early in the pandemic) would be important to test the utility of this approach for future pandemics.

 Despite these limitations, our findings do indicate that adjusting for number and type of LTCs does not substantially reduce the estimated YLL compared to the standard approach. Our analysis does not, however, offer a definitive estimation of YLL across all contexts, nor does it necessarily fully adjust for underlying health status. For example, further work based in Scotland has illustrated that the life expectancy in care-home residents, and therefore the estimated YLL, is substantially different from the general population. This is important given the large proportion of COVID-19 deaths that have occurred in care homes. Additionally, it indicates that additional factors are likely to influence underlying health status, life expectancy, likelihood of dying from Covid-19, and by extension YLL. These factors are not fully represented by the presence or absence of specific LTCs. Some of these factors are likely to be challenging to estimate from routine data alone, and producing YLL estimates which account for these factors should be an area of future investigation.”

***Reviewer:*** *Clarifications to previous comments:*

**Author:**

 Thank you for the clarifications below, and our apologies for the previous misinterpretation. In short, we agree with the points now clarified and have incorporated these into the revised text for the limitations section given above. We highlight the changes in response to the specific points below.

**Reviewer comment:**

*“Author response. Thank you for these comments. Taking first the issue of using data from patients who have died to estimate YLL…it is necessary to use data from people who have died…” - The issue is not taking ‘people who have died’, of course data on deaths is required to estimate YLL (thank you for pointing this out). The issue is, as I alluded to in other comments, if all (or half, more reasonably, even) the people who died were in a care home, as an example, the life expectancy of this selected group is very different from a general population of the same age (median, around 20 months has been estimated, although data is not great - https://www.pssru.ac.uk/pub/3211.pdf). You need to be able to say something about observables (co-variates) of the population of people in your death dataset compared to observable of deaths (/life expectancy) in the general population (especially when the deaths and the life expectancy are from completely different countries/contexts). That is the potential selection effect I mentioned, i.e. it is likely hiding a lot of other confounding. From your response, though, it seems like you are trying to demonstrate what is possible to estimate with flawed data at the start of a pandemic, which is fine. In which case, I would expect this selection effect to be picked up in the limitations (like you have now incorporated in response to my other comments).*

**Author response**

 Thank you for this clarification. The additional text in the abstract conclusion highlights the need to address residual confounding (as you rightly raise here). We also agree about the selection effect and expand on this in the following (also quoted above in fuller context):

 “Our analysis does not, however, offer a definitive estimation of YLL across all contexts, nor does it necessarily fully adjust for underlying health status. For example, further work based in Scotland has illustrated that the life expectancy in care-home residents, and therefore the estimated YLL, is substantially different from the general population. This is important given the large proportion of COVID-19 deaths that have occurred in care homes. Additionally, it indicates that additional factors are likely to influence underlying health status, life expectancy, likelihood of dying from Covid-19, and by extension YLL.”

**Reviewer comment:**

**

*“The standard approach to YLL typically uses just age and sex. We develop this further to include comorbidity, to address the specific point raised in the context of COVID-19, that because of common comorbidities most people who died with COVID-19 would have died soon in any case”. Yes, but adding ‘comorbidity’ in this way does not pick up the severity (proxied by care home/deprivation and other observables I mentioned). Sex and age are largely more straight-forward data points, but co-morbidity measurement is not (there is a lot of literature on this).*

**

**Author response**

 We agree with the reviewer that adjusting for underlying health status is complex and challenging and that an adjustment for the presence/absence of some specific comorbidities does not fully capture that (as proxied by care home status, etc, as the reviewer rightly points out). We highlight this point in the new text quoted in the comment above.

**Reviewer comment**

*“By definition, YLL cannot be observed, neither by hospital data, nor by controlled experiment…”. Again, thank you for pointing this out. But it can, of course, be estimated based on (either older or more updated) data, so it can be ‘observed’ in that sense. What I was getting at with this comment was similar to the above, if around half of the excess deaths are care home residents in most of Europe, and severe co-morbidity is frequently observed, then the YLL you estimate does not intuitively ‘fit’ with these additional observations from the existing literature (it might! But, it needs more work to validate first, I think. As you say, the YLL likely reflects a sub-population of non-care home/without severe conditions – potentially, the YLL in a system, Italy, having suffered long-term austerity and where the health system is overwhelmed). This is where I was getting at the need for some sort of validation in terms of latterly available (superior) data.*

**

**Author response:**

 Thank you for clarifying this point. We agree that the YLL is likely to also reflect underlying factors of the population and health system etc. We have added the following text (again repeated from the longer section above) to highlight this point:

 “While this synthesis of data sources allowed an estimation to be generated at an early stage, it limits the generalisability to specific contexts. Summaries of YLL relating to a specific country or context should ideally use data (both life-expectancy and Covid-19 related) from that context. A comparison of such estimates (based on individual-level and country specific data) with our approach (modelling aggregate- and individual-level data from multiple sources early in the pandemic) would be important to test the utility of this approach for future pandemics.”

**Reviewer comment:**

*“We would also note that none (to our knowledge) have attempted to model the observed events in care homes”. - Comas-Herrera et al. ~42% recorded deaths on average, higher (around half) in earlier parts of epidemic1. Reference 2[ref-] - excess care home mortality in England, ~55% of PHE estimated excess mortality in general population over same period.*

**Author response**

 Thank you. We have added these references along with another estimation of life-expectancy and YLL in care-home residents compared to the general population to the discussion section where the issue of care-homes is discussed.

**

**Reviewer comment**

*“life expectancy at birth. Once an individual is known to have survived to an older age, their life expectancy is much higher than it is at their birth”. Yes, but on average in population, not for most deprived/severely multimorbid/care homes, for instance.*

**

**Author response**

 We agree these are additional important sources of potential confounding, and have added to the abstract and discussion sections as quoted in response to comments above.

**Reviewer comment**

*“Rather we calculate years of life lost for a given set of observed deaths. Since it is not possible to observe years of life lost we are not able to present an observed versus expected comparison”. Yes, but there is now better data on this, even from the same period you analyse. How does it compare to the updated set(s) of ‘observed deaths’?*

**

**Author response**

 We discuss this at greater length in response to the second comment above. In summary, we agree that comparison of our estimate to ‘context-specific’ estimates based on individual level-data will be important particularly if this method for dealing with limited data is to be utilised in future settings. As we discuss above, and in our previous response, we believe such a comparison is beyond the scope of this manuscript:

 “Summaries of YLL relating to a specific country or context should ideally use data (both life-expectancy and Covid-19 related) from that context. A comparison of such estimates (based on individual-level and country specific data) with our approach (modelling aggregate- and individual-level data from multiple sources early in the pandemic) would be important to test the utility of this approach for future pandemics.”

**Reviewer comment**

*“To summarise, we think that this YLL can reasonably be generalised to high-income countries…”. Why is that reasonable? The Covid death profile in UK/Germany/Sweden/Denmark/etc. is completely different, isn’t it? Dependent on some combination of context-specific pre-existing conditions (e.g. ‘stock’ of health and risk factors in the population/resilience and capacity of services to respond) and reactive policy measures, surely?*

**Author response**

 This is a very reasonable point as we have added to the limitation section to highlight this:

 “While this synthesis of data sources allowed an estimation to be generated at an early stage, it limits the generalisability to specific contexts. Summaries of YLL relating to a specific country or context should ideally use data (both life-expectancy and Covid-19 related) from that context.”

**

**Reviewer comment**

*“However, since ten years is already rather a long period, we think that the former is the more pertinent for policy-making”. The more pertinent for policy-making should be the “true” value, surely (i.e. best available estimate given available up-to-date data). Would need to further demonstrate this with validation checks.*

**

**Author response**

 We agree that policy decisions should be based on best available data and using analytic approach that limits bias and confounding. While we demonstrate that

*“This conclusion has been built-upon via subsequent work by other[DM2]  researchers: [REFs]…” As far as I can see, none of these references estimate YLL accounting for multimorbidity or other relevant factors, though, so do not directly support the findings.*

**Author response:**

 This is a fair point; we did not mean to claim that these studies directly support our findings with respect to multimorbidity. However the overall estimates we give are broadly in keeping with subsequent attempts to model YLL. The challenge of ‘other relevant factors’, as the reviewer points out, is important as highlight in response to other comments.

**Reviewer comment:**

*“New data may show that there are other reasons (e.g., high levels of frailty) why the YLL is, after all, considerably lower for deaths from COVID-19 than the age, sex and comorbidity specific estimates suggest”. Personally, I would interpret the care homes literature/all of the literature on individual-level risk factors associated with mortality as already suggesting this. But, again, it’s an empirical question, would be great to see.*

**Author response:**

 This is a reasonable interpretation, as discussed above in response to other comments. The following additional text from the discussion (also quoted above) speaks to this:

**“**Our analysis does not, however, offer a definitive estimation of YLL across all contexts, nor does it necessarily fully adjust for underlying health status. For example, further work based in Scotland has illustrated that the life expectancy in care-home residents, and therefore the estimated YLL, is substantially different from the general population. This is important given the large proportion of COVID-19 deaths that have occurred in care homes. Additionally, it indicates that additional factors are likely to influence underlying health status, life expectancy, likelihood of dying from Covid-19, and by extension YLL. These factors are not fully represented by the presence or absence of specific LTCs.”

# Peer review: Reviewer response for version 2

**DOI:** 10.21956/wellcomeopenres.18306.r42513

**Published:** 2021-02-09

**Authors:** Ramon Martinez-Piedra

**Affiliations:** 1 Department of Non-Communicable Diseases and Mental Health, Pan American Health Organization, Washington, DC, USA

Thanks to authors for this revised version of the article.

Is the work clearly and accurately presented and does it cite the current literature?

Yes

If applicable, is the statistical analysis and its interpretation appropriate?

I cannot comment. A qualified statistician is required.

Are all the source data underlying the results available to ensure full reproducibility?

Yes

Is the study design appropriate and is the work technically sound?

Yes

Are the conclusions drawn adequately supported by the results?

Yes

Are sufficient details of methods and analysis provided to allow replication by others?

Yes

Reviewer Expertise:

Epidemiology, public health, health metrics, data anlysis.

I confirm that I have read this submission and believe that I have an appropriate level of expertise to confirm that it is of an acceptable scientific standard.

# Peer review: Reviewer response for version 1

**DOI:** 10.21956/wellcomeopenres.17385.r41111

**Published:** 2020-11-26

**Authors:** Mei Sum Chan

**Affiliations:** 1 University of Oxford, Oxford, UK; 2 University College London, London, UK

Thank you for the opportunity to review this manuscript. This review relates to the version of the manuscript submitted for review (v1) and does not cover the addendum as the addendum was not mentioned in this version, nor does it refer to the authors’ responses to comments on the manuscript. The review also takes into account that this version was posted on 23 April 2020 and hence did not have access to the better quality data and results from similar studies that were published after this date.

 Overall:

 This is a highly topical investigation into the demographic impact of mortality from COVID-19 and the role of underlying conditions in these mortality patterns. The message that there is a large direct mortality burden from COVID-19, measured in terms of YLL, and that this the burden increases with number of underlying conditions, is clearly made by the study, particularly through results from the first approach (the standard WHO life table approach) and sensitivity analyses of the second approach. However, insufficient evidence was provided on the validity of the second and main approach, a novel complex simulation study of COVID-19 mortality in people with different LTC profiles, both in terms of the statistical rigour and the use of health data within the model. I recommend that substantial model development is carried out to resolve statistical issues and to assess model diagnostics, preferably in the separate methodological investigation that the authors mentioned, before being used in its final form in this empirical study to make policy recommendations.

 The majority of the sections were clear, and compelling reasons were given for conducting this research and for using these results to inform policy and public dialogue. The use of a rapid review to acquire and assess data on underlying conditions is also commendable. The aims were not fully clear and did not match the rest of the manuscript: a target or reference population was not specified, no results by type of multimorbidity were reported even though they were accounted for in the model, and the manuscript focused on the design and application of the novel second approach when this was not a stated aim. The methods, while detailed, were also unclear for the second approach: multiple datasets, model descriptions and procedures were interweaved in no particular order in each subsection, it was difficult to differentiate the role of data vs assumptions, and apart from scenario testing, no model diagnostic or checking procedures were mentioned for the Bayesian components. Some key results on the LTC model convergence and posterior distribution were reported in the Discussion rather than the Results section. One of the conclusions did not appear to match the results well.

 The authors have provided the non-IPD data, annotated code and codelists for this analysis, and given details on the access request procedures for the IPD datasets. Display items for modelled distributions and YLLs were useful but were difficult to interpret for survival curves (Figure 3). A summarised version of Figure 3 would be more useful.

 My key concerns were:

1. The authors reported in the Discussion section that the LTC model in the second approach ‘did not fully converge’. This is a key statistical issue should have been reported much earlier in the manuscript and steps taken to ensure model convergence at the analysis stage. No model diagnostics (for model selection, convergence or goodness of fit) were reported for the LTC model and age model components. Therefore I have major concerns over the validity of the model structure and its results. I also wonder how the authors managed to extract results from the LTC model and how these results should be interpreted since the model was not optimised. The authors’ expectation that the results will not change substantially with future modifications to achieve convergence is not sufficient. If the model lacked input data, why were influenza rather than COVID-19 deaths (specifically the influenza deaths in 2011; Methods section) used to supplement the age model but not the LTC model? What quantity of data or correlation structure in the data was required to enable the model to converge? Is there a way to jointly model the LTC joint prevalence and age distribution? The authors should consider placing more emphasis on the first approach or the simpler sensitivity analyses, or eliminating features of the model that exacerbated non-convergence or may have led to overspecification in this study. Hopefully this would also reduce the reliance of the model on distributional assumptions input by the authors.
2. The abstract described little of the data and methods used for the second approach. The source of data was stated as ‘UK healthcare data’, which was somewhat misleading when in fact only a combination of Scottish and Welsh health records were used and were mixed with aggregate level Italian death data. I could not detect the use of statistical models from reading the abstract, much less a simulation study using a combination of two Bayesian models and one parametric survival model.
3. It was not clear which population the authors intended to model or make recommendations for. This was not stated in the aims, results or discussion, but the choice of data appeared to have a UK focus. The authors have used Scottish, Welsh and Italian data mixed together – presumably these were the datasets that were available to the authors in that short window. However, which population(s) would the results relate to? If only Italian data on underlying conditions was identified in the rapid review, why not focus on Italian data for the rest of the analysis for consistency? The choice of country matters as patterns in multimorbidity and mortality with multimorbidity depend on care provision and demographic characteristics (Nunes et al 2016). The results would also be more informative if country-level differences in mortality profiles at ages 50+ in Italy, Scotland and Wales did not contribute to the reported YLLs.
4. Additionally, the populations used in each model component (Figure 1) were mismatched. The LTC and age models were based on deaths from COVID-19 and/or influenza and their LTC profile at the time of death in several datasets, while the survival model was based on the unselected SAIL population and their LTC profile at baseline. These choices were reasonable within each model component, but inconsistent when these components were combined. The choice of the complex LTC and age model design appeared to be a consequence of using sparse data (deaths from COVID-19 and/or influenza). It would be useful to assess whether the correlation structures of age and LTCs of those who died from these causes were substantially different from the structures of their respective wider populations.
5. Since the authors reported in the Discussion section that the LTC model ‘had wide posteriors (indicating substantial uncertainty)’, the rationale for using a complex LTC model component as part of the main model in addition to the two extreme scenarios of independent LTCs and highly correlated LTCs is unclear. Running the model using those two scenarios alone was sufficient to provide a range of YLLs that would still address the aims of this study and avoid many of the issues raised in this review. In fact, Table 1 shows that the ranges of YLLs described by these two scenarios were not large and the YLLs were substantially larger than zero when age and multimorbidity counts were treated as associated.
6. The authors’ second conclusion that ‘adjustment for number and type of LTCs only modestly reduces the estimated YLL due to COVID-19 compared to estimates based only on age and sex’ seems to gloss over the more policy-relevant finding that there were substantial differences in YLLs by multimorbidity count (Table 2).

Minor comments:

1. Please refer to the relevant citation for ‘age distribution of COVID-19 deaths in Italy from published data’ in the WHO standard YLL approach subsection.
2. Please spell out the first mentions of ‘IPD’ and ‘SAIL’, and move the description of SAIL to the first mention.
3. Please specify what the ‘high correlation’ scenario is rather than the procedure used to obtain it (I think this scenario is specified more clearly in the R code rather than the main text.) The ‘independent’ scenario probably does not require explanation, but if explained, the word ‘random’ should be added.
4. The authors’ comparisons with YLLs from a ‘UK report’ for pneumonia was useful, and vital in the absence of LTC model and age model diagnostics. However, this ‘UK report’ only contains YLLs for England and Wales and not the whole of UK. Published YLLs for other flu pandemics or outbreaks could also be more relevant comparisons.
5. It should be acknowledged that the analysis does not take into account COVID-19 deaths at ages under 50 years, which were rare but may have a non-negligible impact on the summary YLLs for the second approach.
6. The authors refer to the same Github repository for all supplementary material and appendices, which contains a large number of files, mostly datasets, codelists, code and raw output, and no explanation of its contents. It would be helpful to report the interpretations of the outputs and to refer to specific files in the repository in each instance.
7. The estimation of YLLs by type of LTCs was alluded to in the aims but not reported. Some results on YLLs for particular combinations, perhaps the most prevalent combinations, would be more clinically informative than the YLLs by multimorbidity counts. Alternatively, a ranking of LTCs by their lethality or a combined measure of lethality and prevalence would be informative too.
8. The colour coding in Figure 3 was not explained (but is presumably the same as the other figures).
9. The resolutions of the figures could be improved so that the axis labels are more readable.
10. The caption for Table 2 should be revised to clarify that YLLs are tabulated by LTC count and not type.
11. The authors acknowledged that YLL for COVID-19 is an ‘imperfect’ measure, but did not explain why. It would be helpful to add limitations of either YLL approach (eg the YLL models used here do not allow for competing risks) or consider alternative health metrics (eg YLD or DALY) in the Discussion section.
12. The ‘orders of magnitude suggested by some commentators’ mentioned in the Strength and limitations section should cite the relevant reference. Evidence of suggestions or claims by multiple commentators rather than a single commentator would also be appreciated.
13. The Discussion section focused on YLLs by number of LTCs and did not consider the wider context of multiple interlinked risk factors for COVID-19 deaths, several of which are available in the SAIL data (socioeconomic and health behavioural factors). The relative importance of age, sex and number of LTCs (and types of LTCs) should be given some consideration too.
14. The YLL terminology is correct, but it may be worth clarifying (especially to readers who are unfamiliar with YLL or epidemiology) that these YLLs estimate the direct impact of COVID-19 deaths rather than the indirect impact of COVID-19-related outcomes.
15. The comparison of raw death counts with YLLs per person as reporting metrics should be refined as the authors and health organisations appear to stand at cross purposes. One key use of raw death counts is to track the evolution of the pandemic in real time, whereas these YLLs do not track time trends or describe the aggregate mortality burden.

I have limited experience with Bayesian models and would recommend that a reviewer with specific expertise in Bayesian modelling in JAGS is invited at the next round to add any further comments on the Bayesian components of the models if these remain in the analyses.

Is the work clearly and accurately presented and does it cite the current literature?

Partly

If applicable, is the statistical analysis and its interpretation appropriate?

I cannot comment. A qualified statistician is required.

Are all the source data underlying the results available to ensure full reproducibility?

Yes

Is the study design appropriate and is the work technically sound?

Partly

Are the conclusions drawn adequately supported by the results?

Partly

Are sufficient details of methods and analysis provided to allow replication by others?

Yes

Reviewer Expertise:

Epidemiology, medical statistics, gerontology.

I confirm that I have read this submission and believe that I have an appropriate level of expertise to state that I do not consider it to be of an acceptable scientific standard, for reasons outlined above.

## References

[1] : Multimorbidity and mortality in older adults: A systematic review and meta-analysis. *Arch Gerontol Geriatr*.67: 10.1016/j.archger.2016.07.008 130-8 10.1016/j.archger.2016.07.008 27500661

# Response

**Published:** 2021-01-29

**Authors:** Peter Hanlon

**Affiliations:** University of Glasgow, UK

Thank you for your review. We have uploaded a revised version of the manuscript based on your comments and those of the other reviewers. Our responses to each of your comments, along with quoted changes to the manuscript text, are shown below.

 Reviewer comments are shown in italics.

**Reviewer comment**

*Thank you for the opportunity to review this manuscript. This review relates to the version of the manuscript submitted for review (v1) and does not cover the addendum as the addendum was not mentioned in this version, nor does it refer to the authors’ responses to comments on the manuscript. The review also takes into account that this version was posted on 23 April 2020 and hence did not have access to the better quality data and results from similar studies that were published after this date.*

*Overall:*

*This is a highly topical investigation into the demographic impact of mortality from COVID-19 and the role of underlying conditions in these mortality patterns. The message that there is a large direct mortality burden from COVID-19, measured in terms of YLL, and that this the burden increases with number of underlying conditions, is clearly made by the study, particularly through results from the first approach (the standard WHO life table approach) and sensitivity analyses of the second approach. However, insufficient evidence was provided on the validity of the second and main approach, a novel complex simulation study of COVID-19 mortality in people with different LTC profiles, both in terms of the statistical rigour and the use of health data within the model. I recommend that substantial model development is carried out to resolve statistical issues and to assess model diagnostics, preferably in the separate methodological investigation that the authors mentioned, before being used in its final form in this empirical study to make policy recommendations.*

**

*The majority of the sections were clear, and compelling reasons were given for conducting this research and for using these results to inform policy and public dialogue. The use of a rapid review to acquire and assess data on underlying conditions is also commendable. The aims were not fully clear and did not match the rest of the manuscript: a target or reference population was not specified, no results by type of multimorbidity were reported even though they were accounted for in the model, and the manuscript focused on the design and application of the novel second approach when this was not a stated aim. The methods, while detailed, were also unclear for the second approach: multiple datasets, model descriptions and procedures were interweaved in no particular order in each subsection, it was difficult to differentiate the role of data vs assumptions, and apart from scenario testing, no model diagnostic or checking procedures were mentioned for the Bayesian components. Some key results on the LTC model convergence and posterior distribution were reported in the Discussion rather than the Results section. One of the conclusions did not appear to match the results well.*

**

*The authors have provided the non-IPD data, annotated code and codelists for this analysis, and given details on the access request procedures for the IPD datasets. Display items for modelled distributions and YLLs were useful but were difficult to interpret for survival curves (Figure 3). A summarised version of Figure 3 would be more useful.*

**Author response:**

 We thank the reviewer for this summary of their assessment. We outline our response to each of their comments in detail below. In summary, we have added text to explain and justify the use of multiple datasets (due to modelling being conducted early in the pandemic in the context of sparce available data). We have redeveloped our model-fitting approach to achieve statistical convergence and include a detailed description of this, along with model diagnostics, in the online appendix.

**Reviewer comment**

*My key concerns were:*

1. *The authors reported in the Discussion section that the LTC model in the second approach ‘did not fully converge’. This is a key statistical issue should have been reported much earlier in the manuscript and steps taken to ensure model convergence at the analysis stage. No model diagnostics (for model selection, convergence or goodness of fit) were reported for the LTC model and age model components. Therefore I have major concerns over the validity of the model structure and its results. I also wonder how the authors managed to extract results from the LTC model and how these results should be interpreted since the model was not optimised. The authors’ expectation that the results will not change substantially with future modifications to achieve convergence is not sufficient. If the model lacked input data, why were influenza rather than COVID-19 deaths (specifically the influenza deaths in 2011; Methods section) used to supplement the age model but not the LTC model? What quantity of data or correlation structure in the data was required to enable the model to converge? Is there a way to jointly model the LTC joint prevalence and age distribution? The authors should consider placing more emphasis on the first approach or the simpler sensitivity analyses, or eliminating features of the model that exacerbated non-convergence or may have led to overspecification in this study. Hopefully this would also reduce the reliance of the model on distributional assumptions input by the authors.*

**Author response:**

 Thank you for these comments. We have revised our model fitting which now converges fully. We thus arrived at very similar multimorbidity estimates to the previous model, and as such the overall findings of the paper are unchanged.

 Model-fitting details are discussed on the online repository, along with all model diagnostics. We summarise briefly here. The multimodality of the posterior and extreme flexibility of our original model led to poor convergence. By constraining the model a small amount through 1) soliciting more informative priors that led to fewer samples being rejected during sampling, 2) changing how the correlation matrix between conditions was sampled during MCMC fitting to sample whole matrices rather than individual elements which is much more efficient, and 3) treating the absence of a diagnosis in the individual patient data as absence of the disease at a clinically significant level (as all the cases were reviewed by medical teams at the ISS to this standard) we were able to dramatically improve MCMC mixing and satisfy established convergence diagnostics.

 The previous model maximised flexibility (in terms of joint probabilities of LTCs) and propagated this uncertainty throughout the final estimates. The updated model sacrificed some of this flexibility while still propagating uncertainty which facilitated model convergence. Both approaches result in similar estimates, and are based on all available data.

**Reviewer comment**

*The abstract described little of the data and methods used for the second approach. The source of data was stated as ‘UK healthcare data’, which was somewhat misleading when in fact only a combination of Scottish and Welsh health records were used and were mixed with aggregate level Italian death data. I could not detect the use of statistical models from reading the abstract, much less a simulation study using a combination of two Bayesian models and one parametric survival model.*

**Author response:**

 Thank you. We have added detail to the abstract, within the word limits of the journal, to highlight the variety of data sources and modelling strategies employed.

**Reviewer comment**

*It was not clear which population the authors intended to model or make recommendations for. This was not stated in the aims, results or discussion, but the choice of data appeared to have a UK focus. The authors have used Scottish, Welsh and Italian data mixed together – presumably these were the datasets that were available to the authors in that short window. However, which population(s) would the results relate to? If only Italian data on underlying conditions was identified in the rapid review, why not focus on Italian data for the rest of the analysis for consistency? The choice of country matters as patterns in multimorbidity and mortality with multimorbidity depend on care provision and demographic characteristics (Nunes et al 2016). The results would also be more informative if country-level differences in mortality profiles at ages 50+ in Italy, Scotland and Wales did not contribute to the reported YLLs.*

**Author response:**

 We think that this YLL can reasonably be generalised to high-income countries, but most likely to hospitalised patients rather than those who died in care homes. We also argue that public health agencies should produce YLL estimates for their own specific settings using the individual-level patient data to which they now have access, as is currently underway in at least one country. We have added a caveat around care homes to highlight that our estimates cannot be extended to this population. The following text has been added to the manuscript:

 “We chose the WHO life tables to allow comparison of the burden of COVID-19 deaths with other conditions in an international context. However, these, unlike many national-level life tables, do not stratify by sex. Furthermore, our subsequent modelling draws upon data from specific setting based on availability early in the pandemic (namely data on COVID-19 deaths from Italy, and life-expectancy estimates based on data from Wales). Therefore… we performed sensitivity analyses using life tables from Italy (2017), United Kingdom (2016-2018) and, for comparison, the United States (2017).”

 “We chose the best data for age, sex and prevalence of LTCs that was available to us at the time of our modelling, but better-quality individual-level data specific to individual countries will yield substantially more reliable estimates. We would suggest that each public health agency should produce country-specific estimates, using the same LTC definitions in those who died as in the reference population and ideally to an agreed international protocol.”

  “It should be noted that these estimates were made early in the pandemic and could not account for specific patterns and events which emerged within the UK. For example, these analyses were performed before the impact of COVID-19 in care homes in the UK became apparent. SAIL contains data on all participants registered with a GP (and so would include care-home residents), however our estimates of life expectancy do not distinguish between people who live in care-homes and those who do not. As such our analyses would not reflect the YLL at a population level where care-homes are disproportionately impacted. Our estimates, given the data sources which were available at the time, are more likely to reflect the YLL of COVID-19 deaths among hospitalised patients.”

**Reviewer comment**

*Additionally, the populations used in each model component (Figure 1) were mismatched. The LTC and age models were based on deaths from COVID-19 and/or influenza and their LTC profile at the time of death in several datasets, while the survival model was based on the unselected SAIL population and their LTC profile at baseline. These choices were reasonable within each model component, but inconsistent when these components were combined. The choice of the complex LTC and age model design appeared to be a consequence of using sparse data (deaths from COVID-19 and/or influenza). It would be useful to assess whether the correlation structures of age and LTCs of those who died from these causes were substantially different from the structures of their respective wider populations.*

**Author response:**

 The age and sex models were based on people who had died from COVID-19 as YLL can only be inferred as a counterfactual based on the characteristics of those who have died with COVID-19.

 The use of influenza death data was simply to obtain a plausible estimate for how strongly correlated multimorbidity would be with age in the context of death. As the reviewer suggests, we have compared the correlation of age and LTCs in these deaths with the population as a whole, and  obtaining similar results (the point estimate for the whole-population lay within the confidence interval for those who died with influenza, although the estimate for the population as a whole is more precise owing to the larger sample size).

 Finally, in order to estimate YLL it was necessary to obtain estimates of life expectancy based on characteristics of people who died of COVID-19. Assessing life expectancy required us to analyse living patients (which we identified from SAIL, and analysed based on LTC profile, as the reviewer points out).

 It is true that these estimates are drawn from a range of different populations, and we have added the following text to provide context for these decisions:

 “We draw upon data sources available in April 2020, as this modelling study aimed to estimate the potential YLL at an early stage in the pandemic, when the impact was emerging. It should be noted, however, that events unfolding throughout the pandemic are likely to impact the YLL. Any estimate, particularly in the context of a pandemic, is dependent on what populations are exposed, and to what extent. Updated estimates, taking account of events which transpired in the UK and beyond, are the subject of ongoing collaborative efforts and we have not attempted to model these. Rather, this manuscript provides a detailed and reproducible quantification of YLL using techniques targeting the specific challenges of estimation at the early stages of a pandemic.”

**Reviewer comment**

*Since the authors reported in the Discussion section that the LTC model ‘had wide posteriors (indicating substantial uncertainty)’, the rationale for using a complex LTC model component as part of the main model in addition to the two extreme scenarios of independent LTCs and highly correlated LTCs is unclear. Running the model using those two scenarios alone was sufficient to provide a range of YLLs that would still address the aims of this study and avoid many of the issues raised in this review. In fact, Table 1 shows that the ranges of YLLs described by these two scenarios were not large and the YLLs were substantially larger than zero when age and multimorbidity counts were treated as associated.*

**

**Author response:**

 Thank you for this comment. As we have discussed above, and in the online GitHub repository, the model now converges. The overall findings of the paper are unchanged based on this new (converged) model.

**Reviewer comment**

*The authors’ second conclusion that ‘adjustment for number and type of LTCs only modestly reduces the estimated YLL due to COVID-19 compared to estimates based only on age and sex’ seems to gloss over the more policy-relevant finding that there were substantial differences in YLLs by multimorbidity count (Table 2).*

**Author response:**

 We think both findings are interesting, but ultimately the wider point is the most relevant for policy. We are happy to amend the conclusion to reflect that both points are important:

 “While media coverage of the pandemic has focused heavily on COVID-19 affecting people with ‘underlying health conditions’, and while the number and type of LTCs certainly influence the life expectancy and YLL for individuals, adjustment for number and type of LTCs only modestly reduces the estimated YLL due to COVID-19 compared to estimates based only on age and sex.”

**Reviewer comment**

*Minor comments:*

*Please refer to the relevant citation for ‘age distribution of COVID-19 deaths in Italy from published data’ in the WHO standard YLL approach subsection.*

**Author response**

 Thank you. We have added the relevant citation.

**Reviewer comment**

*Please spell out the first mentions of ‘IPD’ and ‘SAIL’, and move the description of SAIL to the first mention*.

**Author response**

 Thank you. We have expanded the first mentions of these.

**Reviewer comment**

*Please specify what the ‘high correlation’ scenario is rather than the procedure used to obtain it (I think this scenario is specified more clearly in the R code rather than the main text.) The ‘independent’ scenario probably does not require explanation, but if explained, the word ‘random’ should be added.*

**Author response:**

 The high-correlation scenario was intended to maximise the multimorbidity load at the individual level. We have added the following text “This generated a dataset where individuals with one comorbidity which reduces life expectancy were more likely to have other comorbidities which reduce life expectancy (and vice versa)”

**Reviewer comment**

*The authors’ comparisons with YLLs from a ‘UK report’ for pneumonia was useful, and vital in the absence of LTC model and age model diagnostics. However, this ‘UK report’ only contains YLLs for England and Wales and not the whole of UK. Published YLLs for other flu pandemics or outbreaks could also be more relevant comparisons.*

**Author response:**

 Thank you for this comment. Unfortunately, however, estimates of YLL for influenza in the UK (or elsewhere) are not generally available. We have amended the text to clarify that the estimates mentioned were for England and Wales.

**Reviewer comment**

*It should be acknowledged that the analysis does not take into account COVID-19 deaths at ages under 50 years, which were rare but may have a non-negligible impact on the summary YLLs for the second approach.*

**Author response:**

 We have added the following text to the discussion:

 “To prevent mean inflation through rare deaths in younger people, who only modelled deaths in people over 50 years.”

**Reviewer comment**

*The authors refer to the same GitHub repository for all supplementary material and appendices, which contains a large number of files, mostly datasets, codelists, code and raw output, and no explanation of its contents. It would be helpful to report the interpretations of the outputs and to refer to specific files in the repository in each instance.*

**Author response:**

 Thank you for this comment. We have organised the GitHub repository including the addition of a README file, and have edited the references to this in the text to allow the relevant sections to be more easily located.

**Reviewer comment**

*The estimation of YLLs by type of LTCs was alluded to in the aims but not reported. Some results on YLLs for particular combinations, perhaps the most prevalent combinations, would be more clinically informative than the YLLs by multimorbidity counts. Alternatively, a ranking of LTCs by their lethality or a combined measure of lethality and prevalence would be informative too.*

**

**Author response:**

 This statement refers to the modelling approach where we simulated likely combinations of LTCs among people who had died of COVID-19. Our life expectancy estimates are therefore based on specific combinations of LTCs, which are then aggregated across LTC counts.

 We do not report estimates for specific combinations of LTCs as our YLL calculations are, by their nature, population-based estimates and presenting specific LTC combinations risks could give the impression that these estimates should be applied to individuals.

**Reviewer comment**

*The colour coding in Figure 3 was not explained (but is presumably the same as the other figures).*

**Author response:**

 Thank you, we have added this to the legend for figure 3.

**Reviewer comment**

*The resolutions of the figures could be improved so that the axis labels are more readable.*

**Author response:**

 We shall re-upload the figures with the revised version.

**Reviewer comment**

*The caption for Table 2 should be revised to clarify that YLLs are tabulated by LTC count and not type.*

**Author response:**

 Thank you. We have added the following footnote to the table to clarify this:

 “*Estimates are based on life-expectancy calculates for specific types and combinations of LTCs, which are then aggregated across LTC counts.”

**Reviewer comment**

*The authors acknowledged that YLL for COVID-19 is an ‘imperfect’ measure, but did not explain why. It would be helpful to add limitations of either YLL approach (eg the YLL models used here do not allow for competing risks) or consider alternative health metrics (eg YLD or DALY) in the Discussion section.*

**Author response:**

 We have added the following text to the discussion to expand on this:

 “In additional to reporting YLL, metrics such as excess deaths and quality-adjusted life years are important to fully contextualise the loss of life seen in the pandemic.”

**Reviewer comment**

*The ‘orders of magnitude suggested by some commentators’ mentioned in the Strength and limitations section should cite the relevant reference. Evidence of suggestions or claims by multiple commentators rather than a single commentator would also be appreciated.*

**Author response:**

 Thank you, we have added citations to this section.

**Reviewer comment**

*The Discussion section focused on YLLs by number of LTCs and did not consider the wider context of multiple interlinked risk factors for COVID-19 deaths, several of which are available in the SAIL data (socioeconomic and health behavioural factors). The relative importance of age, sex and number of LTCs (and types of LTCs) should be given some consideration too.*

**

**Author response:**

 We have added he following text to the discussion section to highlight this issue:

 “Also, we were not able to adjust our estimates for other factors and exposures (such as socioeconomic status, occupation, smoking, health behaviours) which would have given a more accurate representation of life-expectancy in the absence of COVID-19. Socioeconomic status is a particularly pertinent issue, as it may influence not only outcomes from infection (e.g. through multimorbidity and other risk factors) but also the likelihood of exposure (e.g. higher proportions of occupations for which home-working was not feasible). Since socio-economic status also predicts mortality there is a possibility of residual confounding due to the lack of data on socioeconomic status available for our models. We also only modelled deaths in people over 50, and deaths in younger people, while rare, are likely to result in high YLL.”

**Reviewer comment**

*The YLL terminology is correct, but it may be worth clarifying (especially to readers who are unfamiliar with YLL or epidemiology) that these YLLs estimate the direct impact of COVID-19 deaths rather than the indirect impact of COVID-19-related outcomes.*

**Author response:**

 We highlight the issue of direct versus indirect effects in the introduction. We have edited these to emphasise that YLL estimation concerns the direct impact of COVID-19, and have added text to the discussion:

 “These choices will require balancing the likely *direct* effects on mortality from COVID-19 against the likely *indirect* impacts on mortality for other conditions - due for example to inadequate access to necessary services for many people with long term conditions (LTCs), potential reluctance of the public to attend for acute events such as myocardial infarction, or impacts from forced unemployment, loss of income and social isolation. The indirect effects are likely to be complex, most will be downstream, and will require extensive research to be better understood. However, we need to capture the direct effects of COVID-19 as accurately as possible now, via currently available data and methodologies.”

 “Finally, our estimates of YLL only attempt to quantify the direct effects of COVID-19. Indirect impacts on mortality (e.g. through pressure on healthcare services of unintended consequences of lockdown measures) should also be considered, and are not captured by our YLL calculation.”

**Reviewer comment**

*The comparison of raw death counts with YLLs per person as reporting metrics should be refined as the authors and health organisations appear to stand at cross purposes. One key use of raw death counts is to track the evolution of the pandemic in real time, whereas these YLLs do not track time trends or describe the aggregate mortality burden.*

**Author response:**

 We agree that there are limitations to YLL, and that other measures (such as raw death counts, excess deaths etc.) are important to be used alongside. We have added the following to the discussion:

 “In additional to reporting YLL, metrics such as excess deaths and quality-adjusted life years are important to fully contextualise the loss of life seen in the pandemic.”

**Reviewer comment**

*I have limited experience with Bayesian models and would recommend that a reviewer with specific expertise in Bayesian modelling in JAGS is invited at the next round to add any further comments on the Bayesian components of the models if these remain in the analyses.*

**Author response**

 Thank-you for your review.

# Peer review: Reviewer response for version 1

**DOI:** 10.21956/wellcomeopenres.17385.r41112

**Published:** 2020-11-09

**Authors:** Jonathan Stokes

**Affiliations:** 1 Division of Population Health, Health Services Research & Primary Care, University of Manchester, Manchester, UK

Thank you for the opportunity to review a very interesting article exploring the impact of COVID-19 on years of life lost (YLL), adjusting for multimorbid patterns in the population. The topic is clearly highly relevant, and the authors do a good job of explaining how moving beyond the raw numbers of COVID deaths is increasingly important for policymakers, one way of doing so is through YLL. They use a variety of datasets from different countries/contexts to estimate a raw YLL and a multimorbidity-adjusted YLL. The headline figures the authors report are startling, 13 and 11 years for men and women respectively after adjusting for multimorbidity. I do, however, have some major concerns on these estimates, mostly to do with data sources, potential selection effects, and how the reported modelling figure relates to observed actual figures. I would like to see the authors justifying/addressing these prior to recommending indexing. Below I provide more details on the major concerns and then more specific comments on each section. I hope the authors find these useful for strengthening their paper and look forward to seeing the revised version.

 Major comments:

- You draw on a variety of data sources. I’m not entirely convinced it makes sense to put all that you’ve chosen together, though, or that the datasets (especially now) are the best available. You draw on data primarily from Italy for the ‘LTC’ and ‘Age’ models. But Italy is quite a specific context in terms of COVID-19, one where the health system was overwhelmed and, like any other one context, where specific policies were implemented – both of which likely affect the death profile. Is the profile of deaths in this context actually going to be generalisable to any other? The ‘Survival’ model, on the other hand, is from Welsh data. Again, I’m not convinced the mortality profile will be comparable to Italy in ‘normal times’ let alone ‘COVID times’. For example, life expectancy in the UK is 2-3 years less than in Italy and the UK burden of premature death by LTCs is higher than the EU average. The use of WHO life tables is again a potential concern, particularly with no sex-specific details. Life expectancy differs significantly across sex, as does the negative effect of COVID-19. Particularly for the Italian data, the very small numbers are also concerning. You say in the article that at the time this was best available, which makes sense. Surely now there is a better source, though? It would be nice to see all data sources coming from the same country to avoid additional confounding introduced by policy/other context differences. Also, YLL will likely vary significantly by place and over time (e.g. with improvements to policy/treatments and as immunity begins to develop in populations). Making the data sources clearer and more unified would help justify the where/when of the YLL figure you actually present. At the moment it is not at all clear how to interpret the YLL to any context.
- Linked to the above, but probably a more fundamental concern, I wonder if some of the datasets come from a biased population (i.e. selection concerns)? My concern is that both the ‘LTC’ and ‘Age’ models use data from dead patients only, but the ‘Survival’ model then uses all patients with healthcare contacts in Wales. The data sets focusing only on patients who have died can’t be taking into account differences in likelihood of exposure to the virus (for example, more deprived persons with more comorbidities are more likely to be exposed through types of employment and subsequently to die – there are likely going to be different LTC patterns/relationships in the dead versus general population) or severity (for example, a majority of deaths in the UK and other countries are from care home patients – a person with asthma in the general population is going to have a very different life expectancy to someone in a care home with asthma). Can you justify the use of LTC patterns only in patients who have died? On this, I do wonder if there is something you can do with data on those who test positive to at least slightly address this point, perhaps in a two-part model, i.e. first conditioning on exposure to the virus given a set of observables (ideally containing at least deprivation, preferably also setting, e.g. care home), then on subsequent probability of death?
- The YLL figure just doesn’t seem to sit with observed reality. I realise this is a modelling study, but it would be nice to compare your findings to what we have actually observed. For example, what is the average age of death expected from your model compared to observed COVID age of death? Something to contextualise the very high observed average age of death of COVID patients (by some accounts very similar or higher than the average life expectancy for the general population) with the YLL figure you give is needed in the discussion section and/or throughout the results.
- Lastly, I have econometric modelling experience, but not Bayesian. I would recommend to the editorial team to have someone with Bayesian modelling expertise to additionally review the manuscript before a final decision is made.

Specific:

- Introduction
  - I agree with the authors that YLL is an improvement over raw counts of deaths. But, there are also other methods that improve on raw counts, for example excess deaths. It would be good to contextualise/compare the YLL concept with excess deaths, and with concepts such as quality-adjusted life years (the latter might come up again in limitations section also).
  - As above, agree that LTCs and multimorbidity are clearly important considerations for COVID YLL. However, so are other confounders, such as deprivation, etc. Again, would just be nice for some discussion of these other important aspects that are not currently considered in your analysis, either briefly here and/or discussion section.
- Methods
  - Already mentioned in my summary at the top, but the neglect of sex-stratification in the WHO tables seems a major one. Can you justify/look for alternatives?
  - Can you justify the data sources from multiple countries and what the YLL that comes from combining them means? And/or, can you update the datasets to reflect a more coherent, understandable context? The strongest data seems to be the SAIL data you have access to. So, maybe drawing on ONS/CQC data (and life tables too) and/or other publications from the UK context might be a way to do this now? And/or, the US CDC seems to have a lot of data reporting on co-morbidities now, with at least much larger numbers if you still want to try and estimate a ‘global’ YLL estimate.
  - “IPD” (p.4) – spell out
  - For the LTC models, it is not obvious to me how/why you incorporate the Scottish data here. Can you make more clear what this adds and why it is necessary (especially with n=33)?
  - For the age models, why are you using SAIL data on influenza deaths? This is a different disease, so not obvious this is relevant. As above, perhaps there is better data available now?
  - “We arbitrarily chose a standard deviation of 0.5” (p.5). How much influence does this have? Sensitivity analysis?
  - “SAIL is a…” (p.5) You have already introduced SAIL data. Might want to move this up to first introduction.
- Results
  - Throughout, I wondered how the figures you present would compare to the observed. This information should be available now, it would be nice to see validation of the model in this/another way.
  - Table 2: Can you add the absolute number you are expecting to be observed dying in each of these age/MM groups, and again perhaps compare to observed?
- Discussion
  - Make clear what the YLL is exactly, where/when does it relate to? How generalisable?
  - You compare the estimated YLL to established infections/LTCs. Can you say something about likely changes as vaccines/treatments emerge (in light of the billions and billions being spent on them relative to other conditions), and as COVID-19 becomes endemic in populations?
  - The definition of a COVID death varies somewhat across different countries, but tends to be fairly loose (e.g. suspected, or a positive test within X-days but not necessarily the recorded primary cause on the death certificate). Can you discuss implications of using this data, especially in relation to impact of other LTCs? Is it reducing the YLL of other conditions?
  - “As such, the attenuation of YLL following adjustment for LTCs may be an underestimate.” As above, could also be an over-estimate without accounting for exposure to virus/severity of LTCs/different healthy system and time context.
  - “This model did not fully converge and had wide posteriors (indicating substantial uncertainty) for the correlation between LTCs. We nonetheless included the results of this model in our analysis”. This sounds like a pretty big issue. Why is it not converging exactly? What happens when you simplify the model to allow it to converge?
  - “Finally, given the emergent nature of the coronavirus pandemic…”. This paragraph fine for a pre-print, but I think highlights the need for an update now in light of emerging data/evidence.
  - “Among patients dying of COVID-19, there appears to be a considerable burden in terms of years of life lost”. Large proportion of the excess deaths are care home residents. Please discuss severity of disease impact.
  - Quality of life matters too. Need to discuss limitations of YLL and reference important quality of life considerations too.

Is the work clearly and accurately presented and does it cite the current literature?

Partly

If applicable, is the statistical analysis and its interpretation appropriate?

I cannot comment. A qualified statistician is required.

Are all the source data underlying the results available to ensure full reproducibility?

Partly

Is the study design appropriate and is the work technically sound?

Partly

Are the conclusions drawn adequately supported by the results?

Partly

Are sufficient details of methods and analysis provided to allow replication by others?

Partly

Reviewer Expertise:

Research Fellow in Health Economics, PhD, and Master's of Public Health. Expertise in evaluating new models of care for multimorbid patients.

I confirm that I have read this submission and believe that I have an appropriate level of expertise to state that I do not consider it to be of an acceptable scientific standard, for reasons outlined above.

# Response

**Published:** 2021-01-29

**Authors:** Peter Hanlon

**Affiliations:** University of Glasgow, UK

Thank you for your review. We have uploaded a revised version of the manuscript based on your comments and those of the other reviewers. Our responses to each of your comments, along with quoted changes to the manuscript text, are shown below.

 Reviewer comments are shown in italics.

**Reviewer comment**

*Thank you for the opportunity to review a very interesting article exploring the impact of COVID-19 on years of life lost (YLL), adjusting for multimorbid patterns in the population. The topic is clearly highly relevant, and the authors do a good job of explaining how moving beyond the raw numbers of COVID deaths is increasingly important for policymakers, one way of doing so is through YLL. They use a variety of datasets from different countries/contexts to estimate a raw YLL and a multimorbidity-adjusted YLL. The headline figures the authors report are startling, 13 and 11 years for men and women respectively after adjusting for multimorbidity. I do, however, have some major concerns on these estimates, mostly to do with data sources, potential selection effects, and how the reported modelling figure relates to observed actual figures. I would like to see the authors justifying/addressing these prior to recommending indexing. Below I provide more details on the major concerns and then more specific comments on each section. I hope the authors find these useful for strengthening their paper and look forward to seeing the revised version.*

**Author response**

 We thank the reviewer for their comments on this manuscript and are grateful for their recognition of the importance and relevance of the topic.

 We also appreciate that the reviewer highlights areas where the estimates could be improved with current data, particularly around data sources, selection effects, and how these estimates relate to the observed patterns of mortality in the pandemic. We would like to frame our response to these, however, by clarifying what this work is, and what it is not.

 We undertook this work at an early stage in the pandemic (published 23 ^rd^ April) in Europe. At this point, data on deaths in the UK was only just beginning to emerge and was not yet available for analysis. The outbreak in Italy was underway but data on the extent of the impact there remained sparse and poorly resolved (partly, for confidentiality reasons). Our analysis was therefore an attempt to model, at a very early stage in the pandemic and with limited and incomplete data, the impact of COVID-19 on years of life lost (YLL). A major motivation for doing so was to identify whether the high levels of comorbidity among people dying with COVID-19 might mean, as some commentators suggested, that very few years of life were being lost, expressed in the phrase that people dying ‘would have died soon anyway’.

 Our analytic approach was specifically designed and developed to estimate YLL in spite of the sparse data available at the time. It was highly novel in that it used aggregated data on comorbidity to derive estimates of combinations of comorbidity. We did not, and do not, suggest that ours should be the definitive estimate of YLL from COVID-19 more than 6 months later when large quantities of individual-level data from single countries are now available and when the diagnosis and hospital treatment of the disease have undergone such rapid improvement. Indeed, in our original conclusion we suggested that “Public health agencies and governments should report on YLL, ideally adjusting for the presence of underlying LTCs, to allow the public and policy-makers to better understand the burden of this disease”.

 An example of one such effort is a large collaborative effort to calculate YLL in the UK including data on COVID-19 deaths in Wales (SAIL).  This project will not use the specific methods developed for this work, which were designed to estimate YLL adjusting for multimorbidity in the context of sparse and aggregate data (such as in the early stages of a pandemic), but will use simpler methods that are possible when individual-level data are available. Therefore, while the reviewer is correct that various other data-sources could now be utilised to yield estimates of YLL, and that these data would be more complete and more specific to the circumstances of the outbreak in the UK, we do not believe that a revised version of this paper is the place for such an analysis.

 The question, therefore, is whether the work reported via Wellcome Open Research  has – to use the term from the journal’s guidance -- sufficient “academic merit” to meet the standard for publication. We would argue that the current paper has academic merit for three reasons: -

1. We addressed the important clinical/policy question at the time, as to whether the high prevalence of comorbidity/multimorbidity among people dying with COVID-19 meant that the YLL from COVID-19 was substantially lower than the life expectancy conditional on age and sex alone. We demonstrated that it did not, and several other publications have confirmed this conclusion. This was an important finding.
2. We demonstrated that emerging data on the characteristics of people who die during pandemics, even if available only in aggregate form, could be used to robustly estimate years of life lost. We also provided all of the data and code required to implement this method.
3. In doing that, we have developed a novel methodological workflow for inference from *incomplete, sparse data*. This is methodologically a greater challenge than inference from more recent *complete, rich data*. Our analytical approach is neither necessary, nor statistically efficient for very large, patient-level data sets, but will be useful for early-stage estimation in future pandemics.

Therefore, in revising this paper, we have incorporated the reviewer’s comments in two ways:

- Where the concerns are methodological and based on our approach to data available at the early stages of the pandemic, we have updated analyses and amended our results accordingly.
- Where the reviewer highlights data which emerged, or events which transpired, after our initial analysis, we highlight this in the discussion.

We have also rewritten the paper to contextualise the analysis as an attempt to estimate YLL early in the pandemic, with analytical approaches specific to the challenges faced at that time.

 Detailed responses to the comments are given below.

**Reviewer comment**

*Major comments:*

*You draw on a variety of data sources. I’m not entirely convinced it makes sense to put all that you’ve chosen together, though, or that the datasets (especially now) are the best available. You draw on data primarily from Italy for the ‘LTC’ and ‘Age’ models. But Italy is quite a specific context in terms of COVID-19, one where the health system was overwhelmed and, like any other one context, where specific policies were implemented – both of which likely affect the death profile. Is the profile of deaths in this context actually going to be generalisable to any other? The ‘Survival’ model, on the other hand, is from Welsh data. Again, I’m not convinced the mortality profile will be comparable to Italy in ‘normal times’ let alone ‘COVID times’. For example, life expectancy in the UK is 2-3 years less than in Italy and the UK burden of premature death by LTCs is higher than the EU average* *. The use of WHO life tables is again a potential concern, particularly with no sex-specific details. Life expectancy differs significantly across sex, as does the negative effect of COVID-19. Particularly for the Italian data, the very small numbers are also concerning. You say in the article that at the time this was best available, which makes sense. Surely now there is a better source, though? It would be nice to see all data sources coming from the same country to avoid additional confounding introduced by policy/other context differences. Also, YLL will likely vary significantly by place and over time (e.g. with improvements to policy/treatments and as immunity begins to develop in populations). Making the data sources clearer and more unified would help justify the where/when of the YLL figure you actually present. At the moment it is not at all clear how to interpret the YLL to any context.*

**Author response**

 Thank you for these comments. Our choice of data source was based on the best available data at the time. Given that time has now passed and events developed, and continue to evolve, we have added the following to the introduction to clarify that.

 “The SARS-CoV-2 pandemic, the virus causing COVID-19, emerged in late 2019 and continues to have substantial impact on populations and healthcare systems throughout the world. This manuscript presents a revised version of an early analysis – using data sources available in April 2020 - at which time Italy, the first European nation to experience a major outbreak of COVID-19, was seeing rapidly escalating numbers of cases and deaths. In the UK, at that time, the initially small number of hospitalisations and deaths were beginning to rise. The analysis sought to estimate the burden of COVID-19 deaths in terms of potential years of life lost (YLL), at a time when individual-level data on COVID-19 deaths was largely lacking.”

 and

 “We draw upon data sources available in April 2020, as this modelling study aimed to estimate the potential YLL at an early stage in the pandemic, when the impact was emerging. It should be noted, however, that events unfolding throughout the pandemic are likely to impact the YLL. Any estimate, particularly in the context of a pandemic, is dependent on what populations are exposed, and to what extent. Updated estimates, taking account of events which transpired in the UK and beyond, are the subject of ongoing collaborative efforts and we have not attempted to model these. Rather, this manuscript provides a detailed and reproducible quantification of YLL using techniques targeting the specific challenges of estimation at the early stages of a pandemic.”

**Reviewer comment**

*Linked to the above, but probably a more fundamental concern, I wonder if some of the datasets come from a biased population (i.e. selection concerns)? My concern is that both the ‘LTC’ and ‘Age’ models use data from dead patients only, but the ‘Survival’ model then uses all patients with healthcare contacts in Wales. The data sets focusing only on patients who have died can’t be taking into account differences in likelihood of exposure to the virus (for example, more deprived persons with more comorbidities are more likely to be exposed through types of employment and subsequently to die – there are likely going to be different LTC patterns/relationships in the dead versus general population) or severity (for example, a majority of deaths in the UK and other countries are from care home patients – a person with asthma in the general population is going to have a very different life expectancy to someone in a care home with asthma). Can you justify the use of LTC patterns only in patients who have died? On this, I do wonder if there is something you can do with data on those who test positive to at least slightly address this point, perhaps in a two-part model, i.e. first conditioning on exposure to the virus given a set of observables (ideally containing at least deprivation, preferably also setting, e.g. care home), then on subsequent probability of death?*

**Author response**

 Thank you for these comments.

 Taking first the issue of using data from patients who have died to estimate YLL. As the calculation of YLL aims to estimate the potential life expectancy of people dying of a specific cause (under the counterfactual that they had not died of this cause), it is necessary to use data from people who have died. This is a well-established approach for calculating YLL.

 The standard approach to YLL typically uses just age and sex. We develop this further to include comorbidity, to address the specific point raised in the context of COVID-19, that because of common comorbidities most people who died with COVID-19 would have died soon in any case. We agree  however that other factors do influence life expectancy, and agree that in future work, and depending on the exact policy question, variables such as socioeconomic deprivation and ethnicity  could be included.

 We fully agree concerning care homes, however. The high proportion of deaths from COVID-19 occurring among  care-home residents  only emerged after our analysis, as we indicated in an addendum we rapidly published, partly in response to this issue of care homes, 3 weeks after the original publication, and now incorporate into the main manuscript.

 Nonetheless, we strongly agree that care home residents, are a special population, in whom severe disease, multimorbidity and frailty are likely to be commoner. We also agree that there are good biological reasons for suspecting that care home residents may be over-represented among COVID-19 deaths compared to other causes of death (because it is an infectious disease and people in care homes are in a communal residence), and that the inclusion of more care home residents would likely have lowered the YLL in our analyses. Therefore, we would argue that the best approach for determining life expectancy in this group would be to estimate it using data which includes care home residency status ( https://github.com/dmcalli2/covid19_yll_final/blob/master/Scripts/Addendum.md)

  We have now incorporated this point, and the point about socio-economic status into the main manuscript as follows:

 “It should be noted that these estimates were made early in the pandemic and could not account for specific patterns and events which emerged within the UK. For example, these analyses were performed before the impact of COVID-19 in care homes in the UK became apparent. SAIL contains data on all participants registered with a GP (and so would include care-home residents), however our estimates of life expectancy do not distinguish between people who live in care-homes and those who do not. As such, our analyses would not reflect the YLL at a population level where care-homes are disproportionately impacted. Our estimates, given the data sources which were available at the time, are more likely to reflect the YLL of COVID-19 deaths among hospitalised patients.”

 And

 “Socioeconomic status is a particularly pertinent issue, as it may influence not only outcomes from infection (e.g. through multimorbidity and other risk factors) but also the likelihood of exposure (e.g. higher proportions of occupations for which home-working was not feasible).[FC(1]  Since socio-economic status also predicts mortality there is a possibility of residual confounding due to the lack of data on socioeconomic status available for our models.”

 And

 “Additionally, while our estimates do account for multimorbidity, they do not take account of other factors which may impact the likelihood of contracting, or dying from, COVID-19. Socioeconomic status has been found to be an important risk factor for exposure and severity of COVID-19. So too is ethnicity.”

 While the reviewer makes an interesting point about exploring patterns in people who test positive, this is beyond the scope of this manuscript (as described above), particularly as such data did not exist at the time at which these estimates were calculated.

**Reviewer comment**

*The YLL figure just doesn’t seem to sit with observed reality. I realise this is a modelling study, but it would be nice to compare your findings to what we have actually observed. For example, what is the average age of death expected from your model compared to observed COVID age of death? Something to contextualise the very high observed average age of death of COVID patients (by some accounts very similar or higher than the average life expectancy for the general population) with the YLL figure you give is needed in the discussion section and/or throughout the results.*

**Author response**

 By definition, YLL cannot be observed, neither by hospital data, nor by controlled experiment. It can only be inferred as a counterfactual based on the characteristics of those who have died with COVID-19. Age and sex are the most important characteristics, and to these our study has added comorbidity. We agree that additional characteristics should now be added, but such data were not available when we developed our models and, as we have argued, it is beyond the scope of the current project to add these now.

 Moreover, a number of other studies have since attempted to estimate YLL from COVID-19 obtaining estimates have been broadly similar to our own. We would also note that none (to our knowledge) have attempted to model the observed events in care homes (see our qualifications in response to previous point)

 Finally, we would point out that the average life expectancy referred to by the reviewer is the average life expectancy *at birth.* Once an individual is known to have survived to an older age, their life expectancy is much higher than it is at their birth.

**Reviewer comment**

*Introduction*

*I agree with the authors that YLL is an improvement over raw counts of deaths. But, there are also other methods that improve on raw counts, for example excess deaths. It would be good to contextualise/compare the YLL concept with excess deaths, and with concepts such as quality-adjusted life years (the latter might come up again in limitations section also).*

**Author response**

 Thank you. We agree that these other metrics are important and that an assessment of YLL should be viewed alongside these other measures. We have added the following to the discussion:

 “In additional to reporting YLL, metrics such as excess deaths and quality-adjusted life years are important to fully contextualise the loss of life seen in the pandemic.”

**Reviewer comment**

*As above, agree that LTCs and multimorbidity are clearly important considerations for COVID YLL. However, so are other confounders, such as deprivation, etc. Again, would just be nice for some discussion of these other important aspects that are not currently considered in your analysis, either briefly here and/or discussion section.*

**Author response**

 We agree with the reviewer that these are important additional factors. We have added the following text (discussed more fully above):

  “Socioeconomic status is a particularly pertinent issue, as it may influence not only outcomes from infection (e.g. through multimorbidity and other risk factors) but also the likelihood of exposure (e.g. higher proportions of occupations for which home-working was not feasible). There is therefore a likelihood of residual confounding due to the lack of data on socioeconomic status available for our models.”

 And

 “Additionally, while our estimates do account for multimorbidity, they do not take account of other factors which may impact the likelihood of contracting, or dying from, COVID-19. Socioeconomic status and ethnicity have been found to be important risk factors for severity of COVID-19.”

**Reviewer comment** 

*Methods*

*Already mentioned in my summary at the top, but the neglect of sex-stratification in the WHO tables seems a major one. Can you justify/look for alternatives?*

**Author response**

 We have added the following additional analyses to the text:

 Methods:

 “We chose the WHO life tables to allow comparison of the burden of COVID-19 deaths with other conditions in an international context. However, these, unlike many national-level life tables, do not stratify by sex. Furthermore, our subsequent modelling draws upon data from specific setting based on availability early in the pandemic (namely data on COVID-19 deaths from Italy, and life-expectancy estimates based on data from Wales). Therefore, following comments from academic colleagues via social media we performed sensitivity analyses using life tables from Italy (2017), United Kingdom (2016-2018) and, for comparison, the United States (2017).”

 And results:

 “In sensitivity analyses using alternative life tables, life expectancy was lower (particularly for men), however the estimates YLL remained above 10 years for both men and women, regardless of life table used (detailed results shown in https://github.com/dmcalli2/covid19_yll_final/blob/master/Scripts/Addendum.md)”

**Reviewer comment**

*Can you justify the data sources from multiple countries and what the YLL that comes from combining them means? And/or, can you update the datasets to reflect a more coherent, understandable context? The strongest data seems to be the SAIL data you have access to. So, maybe drawing on ONS/CQC data (and life tables too) and/or other publications from the UK context might be a way to do this now? And/or, the US CDC seems to have a lot of data reporting on co-morbidities now, with at least much larger numbers if you still want to try and estimate a ‘global’ YLL estimate.*

**Author response**

 As discussed in detail above, our analysis and approach are based on data sources available early in the pandemic. We have retained this approach but added the following text:

 “To inform our estimates of number and type of LTCs, we first sought to identify the most detailed data available for underlying long-term conditions among people dying of COVID-19. We performed a rapid review…”

**Reviewer comment**

*“IPD” (p.4) – spell out*

**Author response**

 We have changed to “individual patient data”.

**Reviewer comment**

*For the LTC models, it is not obvious to me how/why you incorporate the Scottish data here. Can you make more clear what this adds and why it is necessary (especially with n=33)?*

**Author response**

 This was an issue of statistical identifiability. Estimating joint probabilities (i.e. dependence between comorbidities) from marginal probabilities (aggregated disease counts) alone is an ill-posed problem. Individual-level patient data is much more informative and even sparse noisy individual-level data from Scotland about the correlation between comorbidities was sufficient to break symmetries between otherwise equivalent and hence unidentifiable comorbidity configurations and thereby  improve model convergence.

**Reviewer comment**

*For the age models, why are you using SAIL data on influenza deaths? This is a different disease, so not obvious this is relevant. As above, perhaps there is better data available now?*

**Author response**

 This approach was taken as a compromise given the lack of individual patient data on COVID deaths that was available at the time. We had only aggregate data on LTCs among people who had died, and therefore did not have data on the correlation between age and number of comorbidities. It is possible, but not self-evident, that these would be correlated (as conditioning on COVID-19 death may have influenced the relationship between multimorbidity and age). Therefore, we performed two analyses – assuming no correlation, and assuming correlation between age and multimorbidity. The use of influenza death data was simply to obtain a plausible estimate for how strongly correlated multimorbidity would be with age in the context of death. We accept that influenza and COVID-19 are different diseases, however in the absence of COVID-19 data we had to inform these correlations in some way and so selected a respiratory virus known to cause increased mortality particularly in older people and those with comorbidities. We have added the following to the methods to clarify.

 “While influenza is a different condition, these data were used for the sole purpose of estimating correlations between age and multimorbidity counts (conditioning on death), and did not inform the model in any other way”

**Reviewer comment**

*“We arbitrarily chose a standard deviation of 0.5” (p.5). How much influence does this have? Sensitivity analysis?*

**Author response**

 Increasing the standard deviation to 1 led to an estimated YLL of 13.7 (95% CI 12.7-14.9) for men and 11 (95% CI 10.1-11.9) for women, so it had very little impact on the findings. We have modified the R code in the publicly available repository to allow others to modify the SD as they see fit.

**Reviewer comment**

*“SAIL is a…” (p.5) You have already introduced SAIL data. Might want to move this up to first introduction.*

**Author response**

 Thank you. We have moved this information to the first mention of SAIL.

**Reviewer comment**

*Results*

*Throughout, I wondered how the figures you present would compare to the observed. This information should be available now, it would be nice to see validation of the model in this/another way.*

**Author response**

 We agree this is an interesting and important question. However, as we describe in detail above, this is an entirely distinct methodology to the current manuscript.

**Reviewer comment**

*Table 2: Can you add the absolute number you are expecting to be observed dying in each of these age/MM groups, and again perhaps compare to observed?*

**Author response**

  We do not calculate expected deaths. Rather we calculate years of life lost for a given set of observed deaths. Since it is not possible to observe years of life lost we are not able to present an observed versus expected comparison.

**Reviewer comment**

*Discussion*

*Make clear what the YLL is exactly, where/when does it relate to? How generalisable?*

**Author response**

 In the introduction of the manuscript we set out the definition of YLL:-

 “Within epidemiology, there is a standard measure used to account for this difficulty, the years of *potential* life lost (YLL).(12) YLL can be expressed per-individual who died  as the average number of years an individual would have been expected to live had they not died of a given cause. The conventional approach to YLL uses data on the age at which deaths occurred combined with typical life expectancy at a given age, to estimate a weighted average of the number of years lost. YLL is used to allow fair comparisons of the health impact of different policies – such as different measures to address the pandemic. However, given the controversial role of multimorbidity in COVID-19 deaths it is also important to calculate YLL additionally considering the effects of the presence of a single LTC or multimorbidity”

 The generalisability of the estimate is discussed throughout the manuscript. To summarise, we think that this YLL can reasonably be generalised to high-income countries, but most likely to hospitalised patients rather than those who died in care homes. We also argue that public health agencies should produce YLL estimates for their own specific settings using the individual-level patient data to which they now have access, as is currently underway in. at least one country.

**Reviewer comment**

*You compare the estimated YLL to established infections/LTCs. Can you say something about likely changes as vaccines/treatments emerge (in light of the billions and billions being spent on them relative to other conditions), and as COVID-19 becomes endemic in populations?*

**Author response**

 Thank you for this comment. We have added the following text:

 “It should be noted, however, that YLL for an emergent infection such as COVID-19, particularly in a pandemic, will be sensitive to the specific circumstances of the virus spreading, mitigation strategies, and potential future treatment or vaccines. These estimates, therefore, relate to the specific conditions at the time of modelling and will need to be updated, particularly as vaccination or other strategies alter susceptibility or severity of infection. It is important to note, however, that it would be a misuse of any such modelling if it were used to criticise decision-making undertaken at the time.”

**Reviewer comment**

*The definition of a COVID death varies somewhat across different countries, but tends to be fairly loose (e.g. suspected, or a positive test within X-days but not necessarily the recorded primary cause on the death certificate). Can you discuss implications of using this data, especially in relation to impact of other LTCs? Is it reducing the YLL of other conditions?*

**Author response**

 We agree that in future burden-of-disease estimation studies, where COVID-19 deaths are compared to other causes, difficult decisions will need to be made about the attribution of deaths to different causes such as dementia and COVID-19. However, we think that this is beyond the scope of the current manuscript which was focussed on estimating YLL from COVID-19 while accounting for the age, sex and multimorbidity of those dying with COVID-19.

**Reviewer comment**

*“As such, the attenuation of YLL following adjustment for LTCs may be an underestimate.” As above, could also be an over-estimate without accounting for exposure to virus/severity of LTCs/different healthy system and time context.*

**Author response**

 We agree that the attenuation of YLL (ie the decrement in YLL when life expectancy is calculated using age, sex and comorbidity rather than just age and sex) could (compared to a model containing all predictors of mortality) be over or underestimated due to unmeasured confounding. However, since ten years is already rather a long period, we think that the former is the more pertinent for policy-making.

**Reviewer comment**

*“This model did not fully converge and had wide posteriors (indicating substantial uncertainty) for the correlation between LTCs. We nonetheless included the results of this model in our analysis”. This sounds like a pretty big issue. Why is it not converging exactly? What happens when you simplify the model to allow it to converge?*

**Author response**

 We have revised our model fitting which now converges fully. We thus arrived at very similar multimorbidity estimates to the previous model, and as such the overall findings of the paper are unchanged.

 Model-fitting details are discussed on the online repository, along with all model diagnostics. We summarise briefly here. The multimodality of the posterior and extreme flexibility of our original model led to poor convergence. By constraining the model a small amount through 1) soliciting more informative priors that led to fewer samples being rejected during sampling, 2) changing how the correlation matrix between conditions was sampled during MCMC fitting to sample whole matrices rather than individual elements which is much more efficient, and 3) treating the absence of a diagnosis in the individual patient data as absence of the disease at a clinically significant level (as all the cases were reviewed by medical teams at the ISS to this standard) we were able to dramatically improve MCMC mixing and satisfy established convergence diagnostics.

 The previous model maximised flexibility (in terms of joint probabilities of LTCs) and propagated this uncertainty throughout the final estimates. The updated model sacrificed some of this flexibility while still propagating uncertainty which facilitated model convergence. Both approaches result in similar estimates, and are based on all available data.

**Reviewer comment**

*“Finally, given the emergent nature of the coronavirus pandemic…”. This paragraph fine for a pre-print, but I think highlights the need for an update now in light of emerging data/evidence.*

**Author response**

 For health technology assessments of new interventions (e.g. of a novel drug), we would agree that an interim analysis based on aggregate data would be of limited use and that the decision should wait for a full analysis with all available data. However, as we have outlined more fully in our opening statement, we think that this work does have scientific and policy value as it stands – not least because it specifically addressed the question of whether the high prevalence of comorbidity/multimorbidity *per se* among those dying with COVID-19 should mean that the consequences of deaths from COVID-19 to individuals and society are less than the total rates would imply. Using the available data we showed that this view was not supported by the data. This conclusion has been built-upon via subsequent work by other[DM2]  researchers:

https://www.medrxiv.org/content/10.1101/2020.06.15.20131540v1

https://www.ncbi.nlm.nih.gov/pmc/articles/PMC7499646/

https://www.medrxiv.org/content/10.1101/2020.06.08.20050559v2

https://www.ncbi.nlm.nih.gov/pmc/articles/PMC7513788/

https://www.scielosp.org/article/csp/2020.v36n11/e00148920/en/

https://www.medrxiv.org/content/10.1101/2020.10.24.20218693v2

 New data may show that there are other reasons (e.g., high levels of frailty) why the YLL is, after all, considerably lower for deaths from COVID-19 than the age, sex and comorbidity specific estimates suggest. However, this would be a new finding, beyond the scope of the current work.

**Reviewer comment**

*“Among patients dying of COVID-19, there appears to be a considerable burden in terms of years of life lost”. Large proportion of the excess deaths are care home residents. Please discuss severity of disease impact.*

**Author response**

 Thank you. We agree this is an important issue and have added the following:

 “It should be noted that these estimates were made early in the pandemic and could not account for specific patterns and events which emerged within the UK. For example, these analyses were performed before the impact of COVID-19 in care homes in the UK became apparent. SAIL contains data on all participants registered with a GP (and so would include care-home residents), however our estimates of life expectancy do not distinguish between people who live in care-homes and those who do not. As such our analyses would not reflect the YLL at a population level where care-homes are disproportionately impacted. Our estimates, given the data sources which were available at the time, are more likely to reflect the YLL of COVID-19 deaths among hospitalised patients.”

**Reviewer comment**

*Quality of life matters too. Need to discuss limitations of YLL and reference important quality of life considerations too.*

**Author response**

 Thank you. We agree and have added the following text to the discussion.

 “In additional to reporting YLL, metrics such as excess deaths and quality-adjusted life years are important to fully contextualise the loss of life seen in the pandemic.”

# Peer review: Reviewer response for version 1

**DOI:** 10.21956/wellcomeopenres.17385.r38609

**Published:** 2020-06-08

**Authors:** Ramon Martinez-Piedra

**Affiliations:** 1 Department of Non-Communicable Diseases and Mental Health, Pan American Health Organization, Washington, DC, USA

This study aims to quantify the burden of premature mortality related to COVID-19 using years of life lost as a health-gap measure. It goes further by quantifying premature mortality from COVID-19 based on multi-morbidity or number of underlying long-term conditions.

 This is a relevant topic as the study can provide useful information for planning and prioritizing public health and health care interventions and policies. The methodological approach from this study can serve to further research studies particularly quantifying the impact of multi-morbidity on premature mortality.

 The manuscript is well written, clear, and straight forward.

 I don't have any major criticisms of the study and manuscript.

Is the work clearly and accurately presented and does it cite the current literature?

Yes

If applicable, is the statistical analysis and its interpretation appropriate?

I cannot comment. A qualified statistician is required.

Are all the source data underlying the results available to ensure full reproducibility?

Yes

Is the study design appropriate and is the work technically sound?

Yes

Are the conclusions drawn adequately supported by the results?

Yes

Are sufficient details of methods and analysis provided to allow replication by others?

Yes

Reviewer Expertise:

Epidemiology, public health, health metrics, data anlysis.

I confirm that I have read this submission and believe that I have an appropriate level of expertise to confirm that it is of an acceptable scientific standard.
